# Supplementary material for: Purkinje cell intrinsic activity shapes cerebellar development and function
Source: Nat Commun. 2026 Mar 9;17:3688. doi: 10.1038/s41467-026-70355-w (PMC13100008; doi:10.1038/s41467-026-70355-w)
Supplement: Supplementary file 1 — Supplementary Information [file 41467_2026_70355_MOESM1_ESM.pdf]

Supplementary Information

Purkinje cell intrinsic activity shapes cerebellar development and function

Catarina Osório, Joshua J. White, Paula Torrents-Solé, Jie Yang, Nienke Mandemaker, Federico Olivero, Freya Kirwan, Laura Post, Zahra Hemmat, Fred de Winter, Eleonora Regolo, Francesca Romana Fiocchi, Inês Serra, Saffira Tjon, Zeliha Ozgur, Mirjam C.G.N. van den Hout, Wilfred F. J. van IJcken, Guillermina López-Bendito, Aleksandra Badura, Lynette Lim, Geeske M. van Woerden, Martijn Schonewille

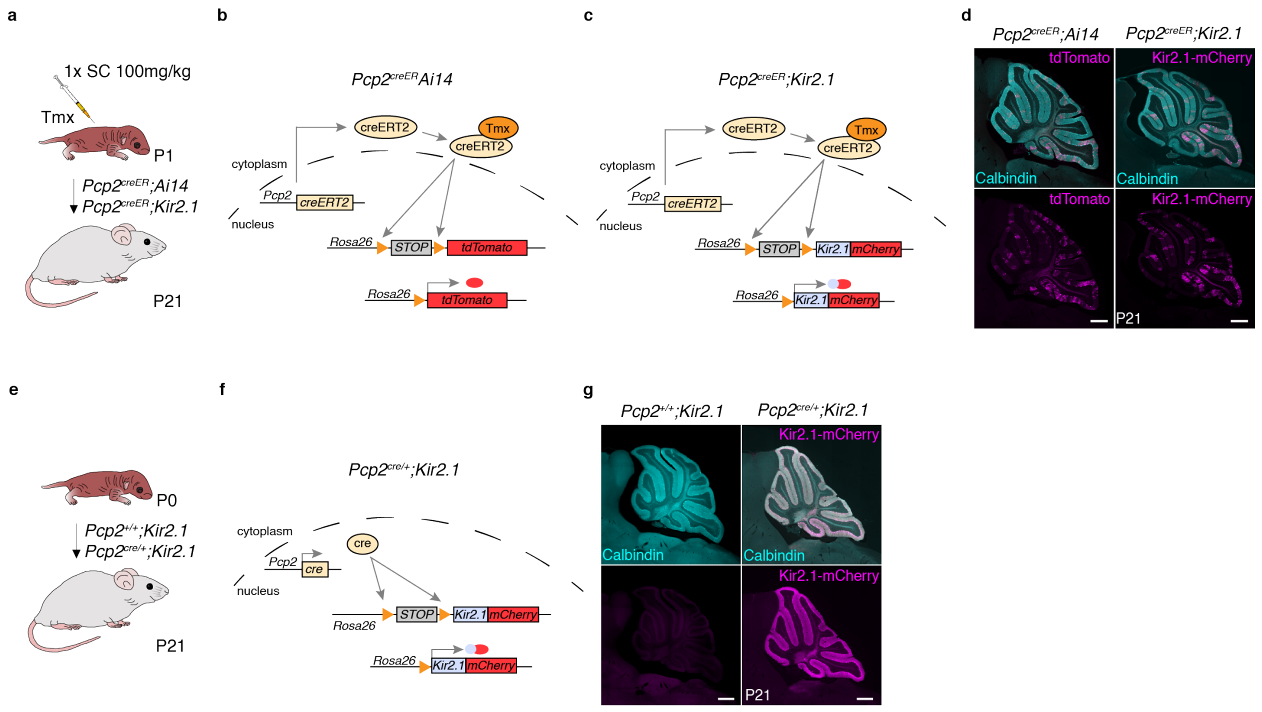

Supplementary Fig. 1 Selective labeling of Purkinje cells at P21 in different mouse models.
a Experimental design. *Pcp2^{creER};Ai14* and *Pcp2^{creER};Kir2.1* pups were injected subcutaneously (SC) with tamoxifen P1 (Tmx, 100mg/kg) at postnatal day 1 (P1), and brains were collected at P21. **b**, **c** Schematics illustrating the genetic strategies used to selectively overexpress tdTomato or Kir2.1-mCherry in Purkinje cells, respectively. **d** Sagittal cerebellar sections at P21 from *Pcp2^{creER};Ai14 and Pcp2^{creER};Kir2.1* mice show sparse labeling of tdTomato (magenta) or Kir2.1-mCherry (magenta), respectively, in Purkinje cells co-labeled with calbindin (cyan). **e** Experimental design. Brains from conditional *Pcp2$^{cre/+}$;Kir2.1* and littermate control *Pcp2$^{+/+}$;Kir2.1* were collected at P21. **f** Schematic illustrating the genetic strategy used to achieve Purkinje cell-specific Kir2.1-mCherry expression. **g** Sagittal cerebellar sections at P21 from *Pcp2$^{+/+}$;Kir2.1* and *Pcp2$^{cre/+}$;Kir2.1* mice show absence or presence of Kir2.1-mCherry (magenta), respectively, in Purkinje cells labeled with calbindin (cyan). *Pcp2*, Purkinje cell protein 2 promoter; cre, recombinase; ERT2, human estrogen receptor ligand-binding domain; *Rosa26*, ubiquitous promoter/enhancer; STOP, sequence interrupting gene expression; tdTomato, red fluorescent protein; Kir2.1, inward-rectifier potassium channel; mCherry, red fluorescent protein. Scale bar, 500 µm (**d**, **g**). Data related to Fig. 1.

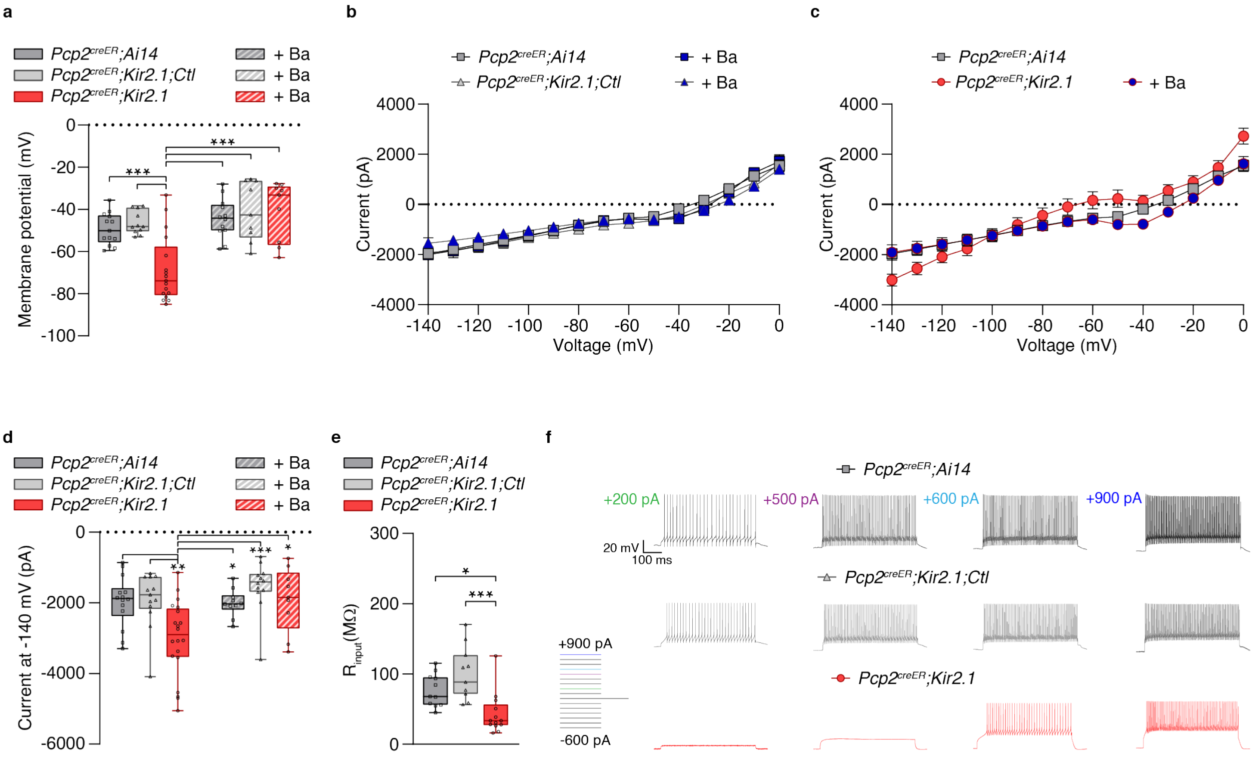

Supplementary Fig. 2 Overexpression of Kir2.1 decreases the excitability of Purkinje cells at P21. **a** Quantification of resting membrane potential in Purkinje cells from *Pcp2*^{creER}*;Ai14* (n = 13 cells/3 mice), *Pcp2*^{creER}*;Kir2.1,Ctl* (n = 11 cells/4 mice) and *Pcp2*^{creER}*;Kir2.1* (n = 17 cells/4 mice), without and with barium (respectively, n = 12 cells/5 mice, n = 9 cells/5 mice, n = 9 cells/5 mice). One-way ANOVA followed by Tukey's multiple comparisons test: ****P* < 0.001. **b** Current-voltage (I-V) relationship from Purkinje cells of *Pcp2*^{creER}*;Ai14* (n = 14 cells/4 mice) and *Pcp2*^{creER}*;Kir2.1,Ctl* (n = 13 cells/5 mice) under normal conditions and in the presence of barium (Ba, 300 μM) at P21 (*Pcp2*^{creER}*;Ai14* + Ba: n = 10 cells/5 mice; *Pcp2*^{creER}*;Kir2.1,Ctl* + Ba: n = 12 cells/4 mice). **c** I-V relationship from Purkinje cells of *Pcp2*^{creER}*;Ai14* (n = 14 cells/4 mice) and *Pcp2*^{creER}*;Kir2.1* (n = 20 cells/6 mice) under normal conditions and in the presence of barium (*Pcp2*^{creER}*;Kir2.1* + Ba: n = 9 cells/3 mice). **d** Quantification of current amplitude at -140 mV in the absence or presence of barium across experimental groups. One-way ANOVA followed by Tukey's multiple comparisons test: **P* < 0.05, ***P* < 0.01, ****P* < 0.001. **e** Quantification of input resistance in Purkinje cells from *Pcp2*^{creER}*;Ai14* (n = 11 cells/3 mice), *Pcp2*^{creER}*;Kir2.1,Ctl* (n = 11 cells/4 mice) and *Pcp2*^{creER}*;Kir2.1* (n = 13 cells/4 mice). Kruskal-Wallis multiple comparisons test: **P* < 0.05, ****P* < 0.001. **f** Representative voltage responses to step current injections (200, 500, 600, and 900 pA) from *Pcp2*^{creER}*;Ai14*, *Pcp2*^{creER}*;Kir2.1,Ctl,* and *Pcp2*^{creER}*;Kir2.1* Purkinje cells at P21. Box plots indicate the median (middle line), 25th and 75th percentiles (box), and 5th and 95th percentiles (whiskers) (**a**, **d**, **e**). Data are shown as the mean ± s.e.m. (**b**, **c**). Statistical details are provided in Supplementary Table 1. Source data are provided as a Source data file. Data related to Fig. 1.

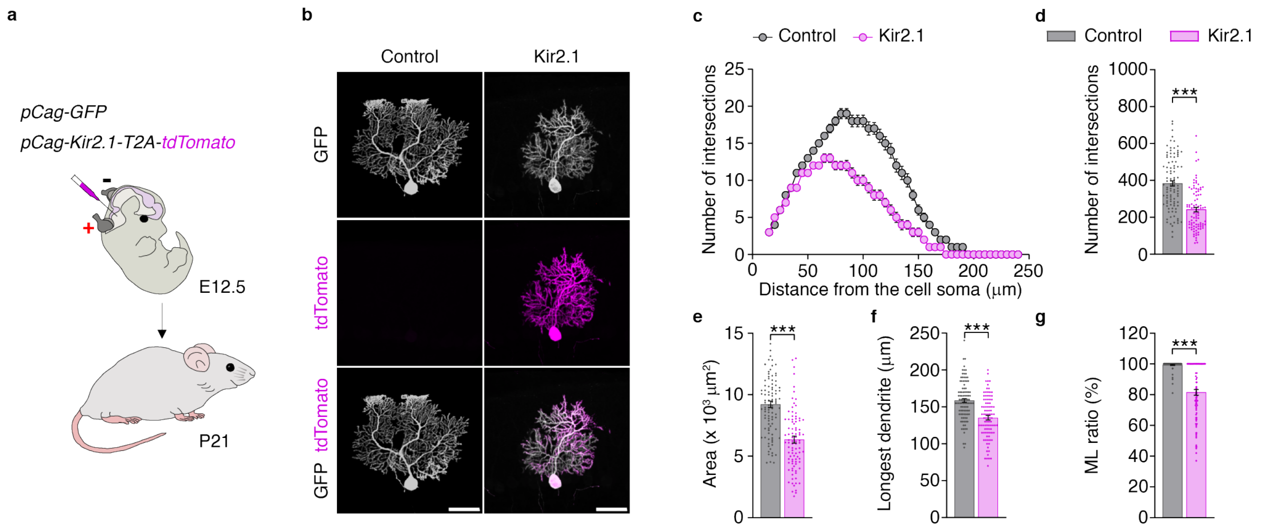

Supplementary Fig. 3 Overexpression of Kir2.1 impairs the development of Purkinje cell dendritic arbor at P21. a Experimental design. *In utero* electroporation of *pCag-GFP* (Control) and *pCag-Kir2.1-T2A-tdTomato* (Kir2.1) plasmids into Purkinje cell progenitors was performed at embryonic day 12.5 (E12.5). Brains were collected at postnatal day 21 (P21). **b** Representative images of Purkinje cell morphology in control (grey) and Kir2.1 (magenta) groups at P21. Scale bar, 50 µm. **c** Quantification of dendrite complexity using Sholl analysis, including **d** number of intersections, **e** cell area, **f** longest dendrite, and **g** molecular layer (ML) ratio in control (n = 98 cells/4 mice) and Kir2.1 (n = 92 cells/4 mice) Purkinje cells**.** Mann-Whitney *U* test and unpaired Student's *t*-test: ***$P < 0.001$. Data are shown as the mean ± s.e.m. Statistical details are provided in Supplementary Table 1. Source data are provided as a Source data file. Cag, CMV early enhancer/chicken beta-actin promoter; GFP, green fluorescent protein; Kir2.1, inward-rectifier potassium channel; T2A, self-cleaving oligopeptide sequence. Data related to Fig. 2.

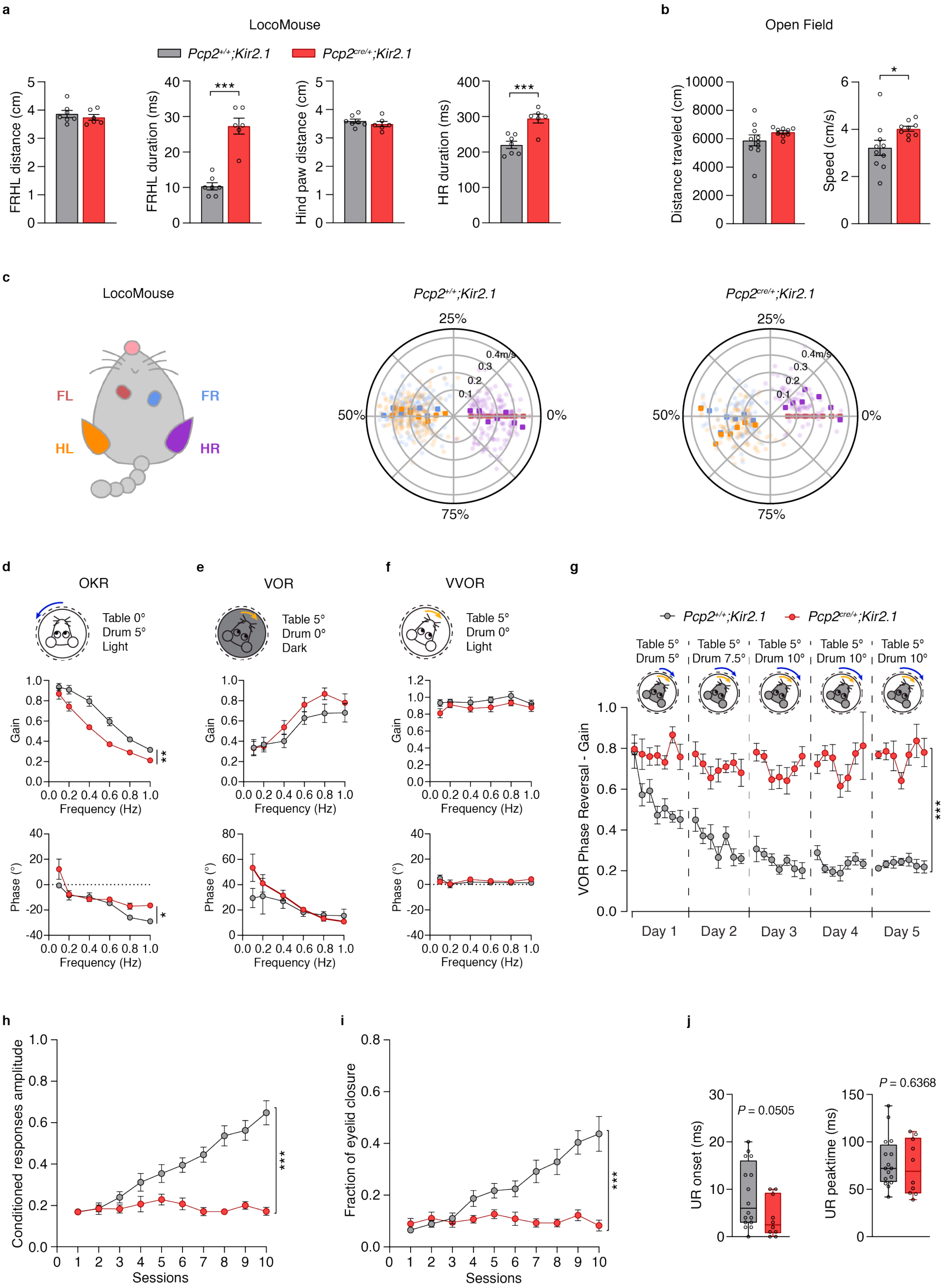
a
LocoMouse
Pcp2+/+;Kir2.1
Pcp2cre/+;Kir2.1
FRHL distance (cm)
FRHL duration (ms)
Hind paw distance (cm)
HR duration (ms)
***
b
Open Field
Distance traveled (cm)
Speed (cm/s)
*
c
LocoMouse
FL
FR
HL
HR
25%
50%
0%
75%
0.4m/s
0.3
0.2
0.1
d
OKR
Table 0°
Drum 5°
Light
e
VOR
Table 5°
Drum 0°
Dark
f
VVOR
Table 5°
Drum 0°
Light
Gain
Phase (°)
Frequency (Hz)
g
VOR Phase Reversal - Gain
Table 5°
Drum 5°
Drum 7.5°
Drum 10°
Day 1
Day 2
Day 3
Day 4
Day 5
h
Conditioned responses amplitude
Sessions
i
Fraction of eyelid closure
j
UR onset (ms)
P = 0.0505
UR peaktime (ms)
P = 0.6368

Supplementary Fig. 4 Reduction of Purkinje cell intrinsic activity impairs motor performance and motor learning in adult mice. a LocoMouse gait parameters, including front-right hind-left (FRHL) paw distance and duration, hind paw distance, and hind-right (HR) duration in *Pcp2$^{+/+}$;Kir2.1* (n = 7 mice) and *Pcp2$^{cre/+}$;Kir2.1* (n = 6 mice). Unpaired Student's *t*-test: ****P* < 0.001. **b** Open field test showing distance traveled and average speed in *Pcp2$^{+/+}$;Kir2.1* (n = 10 mice) and *Pcp2$^{cre/+}$;Kir2.1* (n = 9 mice) groups. Unpaired Student's *t*-test with Welch's correction: **P* < 0.05. **c** Schematic of a mouse with color-coded paws and polar plots showing paw phase in the step cycle relative to the front-left (FL) paw (red). Each radial axis represents a walking speed. HL, hind-left (orange); FR, front-right (blue); HR, hind-right (purple). Quantification of baseline compensatory eye movements, including gain and phase in **d** the optokinetic reflex (OKR), **e** the vestibular-ocular reflex (VOR), and **f** the visually enhanced VOR (VVOR) in *Pcp2$^{+/+}$;Kir2.1* (n = 11 mice) and *Pcp2$^{cre/+}$;Kir2.1* (n = 11 mice). Mixed-effect analysis with repeated measures: **P* < 0.05, ***P* < 0.01. **g** Quantification of VOR gain-reversal training across five days in *Pcp2$^{+/+}$;Kir2.1* (n = 7 mice) and *Pcp2$^{cre/+}$;Kir2.1* (n = 7 mice). Mixed-effect analysis with repeated measures: ****P* < 0.001. **h** Conditioned response amplitude and **i** fraction of eyelid closure during eyeblink conditioning in *Pcp2$^{+/+}$;Kir2.1* (n = 16 mice) and *Pcp2$^{cre/+}$;Kir2.1* (n = 10 mice). Mixed-effect analysis with repeated measures and two-way repeated measures ANOVA: ****P* < 0.001. **j** Onset and peak time of the unconditioned response (UR) in *Pcp2$^{+/+}$;Kir2.1* (n = 16 mice) and *Pcp2$^{cre/+}$;Kir2.1* (n = 10 mice). Mann-Whitney *U* test and unpaired Student's *t*-test. Data are shown as the mean ± s.e.m. except in **j**, where data are presented in box plots indicating the median (middle line), 25th and 75th percentiles (box), and 5th and 95th percentiles (whiskers). Statistical details are provided in Supplementary Table 1. Source data are provided as a Source data file. Data related to Fig. 3.

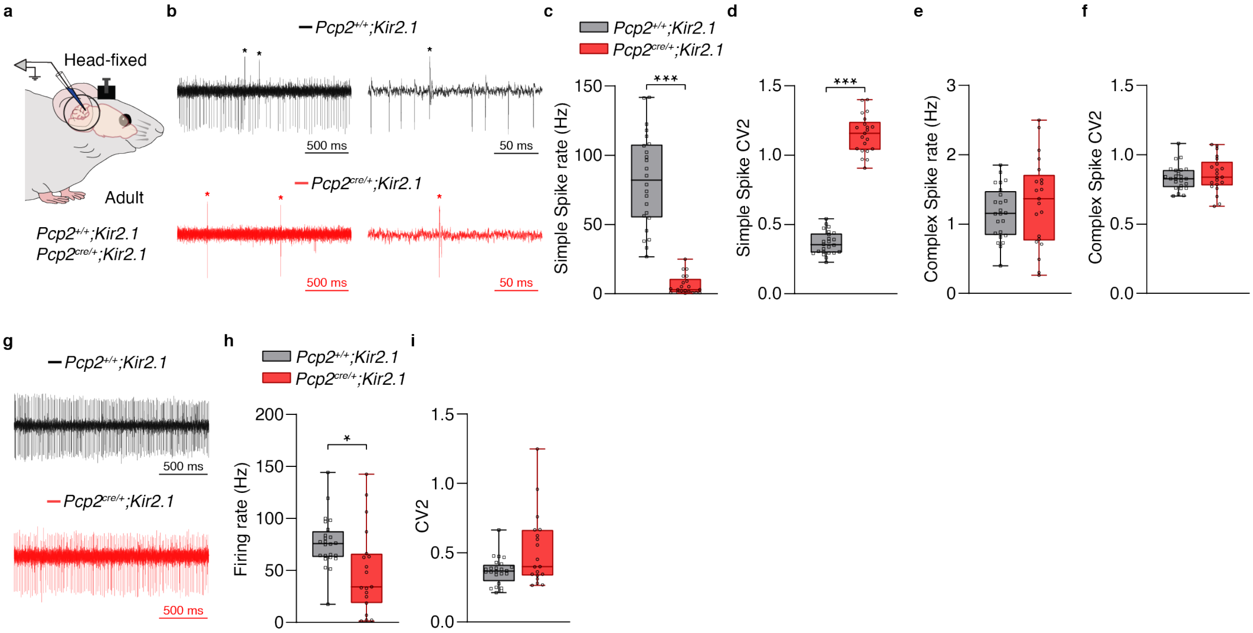

Supplementary Fig. 5 Overexpression of Kir2.1 reduces simple spike activity in adult mice.

a Experimental design. Extracellular recordings were made from Purkinje cells or cerebellar nuclei neurons in awake adult *Pcp2*$^{+/+}$*;Kir2.1* and *Pcp2*$^{cre/+}$*;Kir2.1* mice. **b** Example traces of *Pcp2*$^{+/+}$*;Kir2.1* (black) and *Pcp2*$^{cre/+}$*;Kir2.1* (red) Purkinje cells. Complex spikes are identified with an asterisk. **c** Simple spike firing rate, **d** coefficient of variation 2 (CV2) for simple spikes, **e** complex spike firing rate, and **f** CV2 for complex spikes in Purkinje cells from *Pcp2*$^{+/+}$*;Kir2.1* (n = 24 cells/5 mice) and *Pcp2*$^{cre/+}$*;Kir2.1* (n = 21 cells/4 mice). Mann-Whitney *U* test and unpaired Student's *t*-test with Welch's correction: ***$P < 0.001$. **g** Example traces of *Pcp2*$^{+/+}$*;Kir2.1* (black) and *Pcp2*$^{cre/+}$*;Kir2.1* (red) cerebellar nuclei neurons. **h** Firing rate and **i** CV2 in cerebellar nuclei neurons from *Pcp2*$^{+/+}$*;Kir2.1* (n = 24 cells/5 mice) and *Pcp2*$^{cre/+}$*;Kir2.1* mice (n = 19 cells/4 mice). Unpaired Student's *t*-test with Welch's correction and Mann-Whitney *U* test: *$P < 0.05$. Data are presented in box plots indicating the median (middle line), 25th and 75th percentiles (box), and 5th and 95th percentiles (whiskers). Statistical details are provided in Supplementary Table 1. Source data are provided as a Source data file. Data related to Fig. 3.

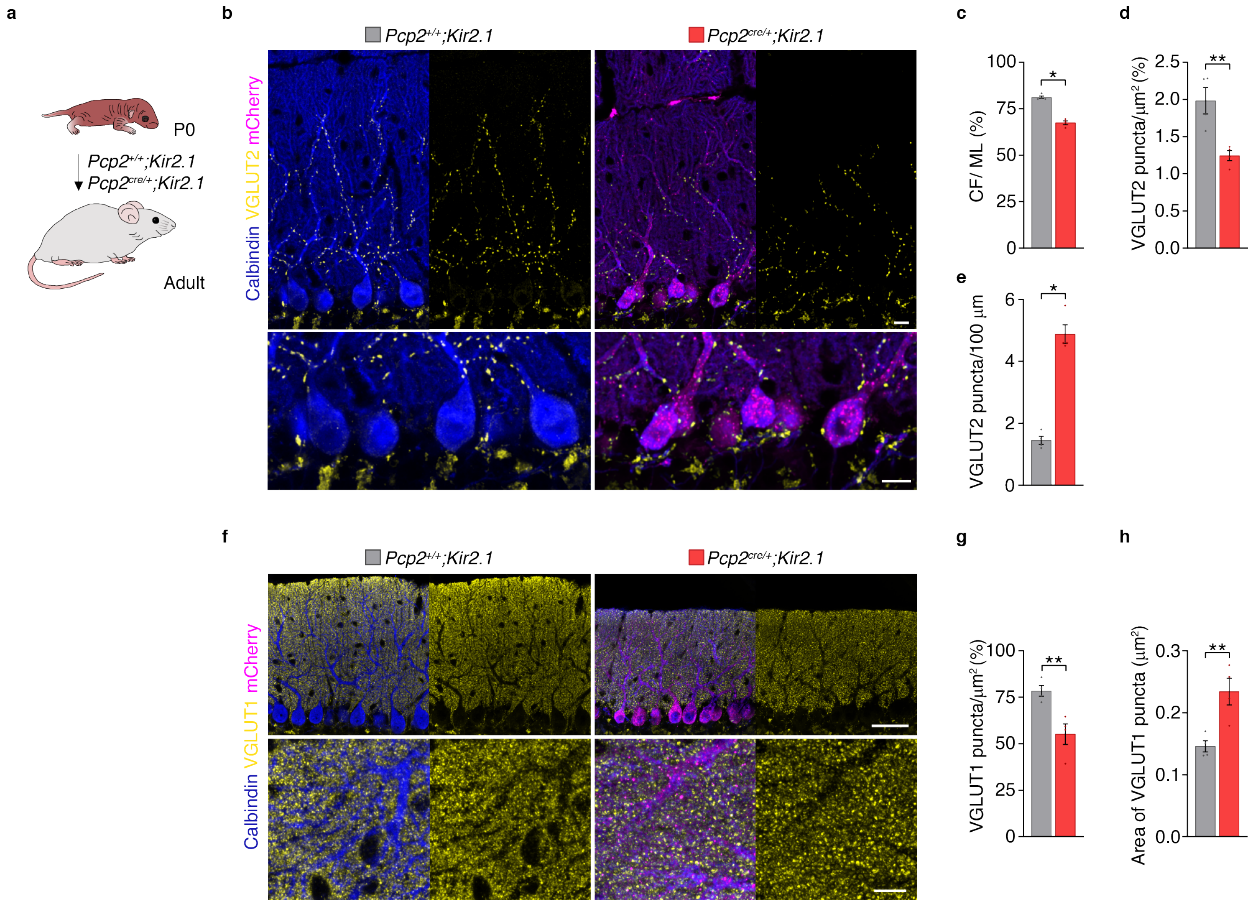

Supplementary Fig. 6 Overexpression of Kir2.1 reduces the number of excitatory VGLUT2 and VGLUT1 presynaptic markers on Purkinje cells dendrites in adult mice. a Experimental design. Brains from *Pcp2*$^{cre/+}$*;Kir2.1* and littermate controls *Pcp2*$^{+/+}$*;Kir2.1* were collected in adulthood. **b** Representative images (top) and high-magnification views (bottom) showing Purkinje cells (blue) and VGLUT2-positive presynaptic boutons (yellow) in *Pcp2*$^{+/+}$*;Kir2.1 and Pcp2*$^{cre/+}$*;Kir2.1* mice (Kir2.1-mCherry expressing Purkinje cells, magenta). **c** Percentage of climbing fiber (CF) extension per molecular layer (ML) thickness, **d** percentage of VGLUT2 puncta per area of region of interest (ROI), and **e** density of VGLUT2 puncta per 100 µm of Purkinje cell soma perimeter in *Pcp2*$^{+/+}$*;Kir2.1* (n = 4 mice) and *Pcp2*$^{cre/+}$*;Kir2.1* (n = 4 mice). Mann-Whitney *U* test and unpaired Student's *t*-test: $^*P < 0.05$, $^{**}P < 0.01$. **f** Representative images (top) and high-magnification views (bottom) showing Purkinje cells (blue) and VGLUT1-positive presynaptic boutons (yellow) in *Pcp2*$^{+/+}$*;Kir2.1 and Pcp2*$^{cre/+}$*;Kir2.1* mice (Kir2.1-mCherry expressing Purkinje cells, magenta). **g** Percentage of VGLUT1 puncta per ROI area, and **h** VGLUT1 puncta area per ROI in *Pcp2*$^{+/+}$*;Kir2.1* (n = 4 mice) and *Pcp2*$^{cre/+}$*;Kir2.1* (n = 4 mice). Unpaired Student's *t*-test: $^{**}P < 0.01$. Scale bars, 10 µm (**b** and bottom panels in **f**); 50 µm (top panels in **f**). Data are shown as the mean ± s.e.m. Statistical details are provided in Supplementary Table 1. Source data are provided as a Source data file. P, postnatal. Data related to Fig. 3.

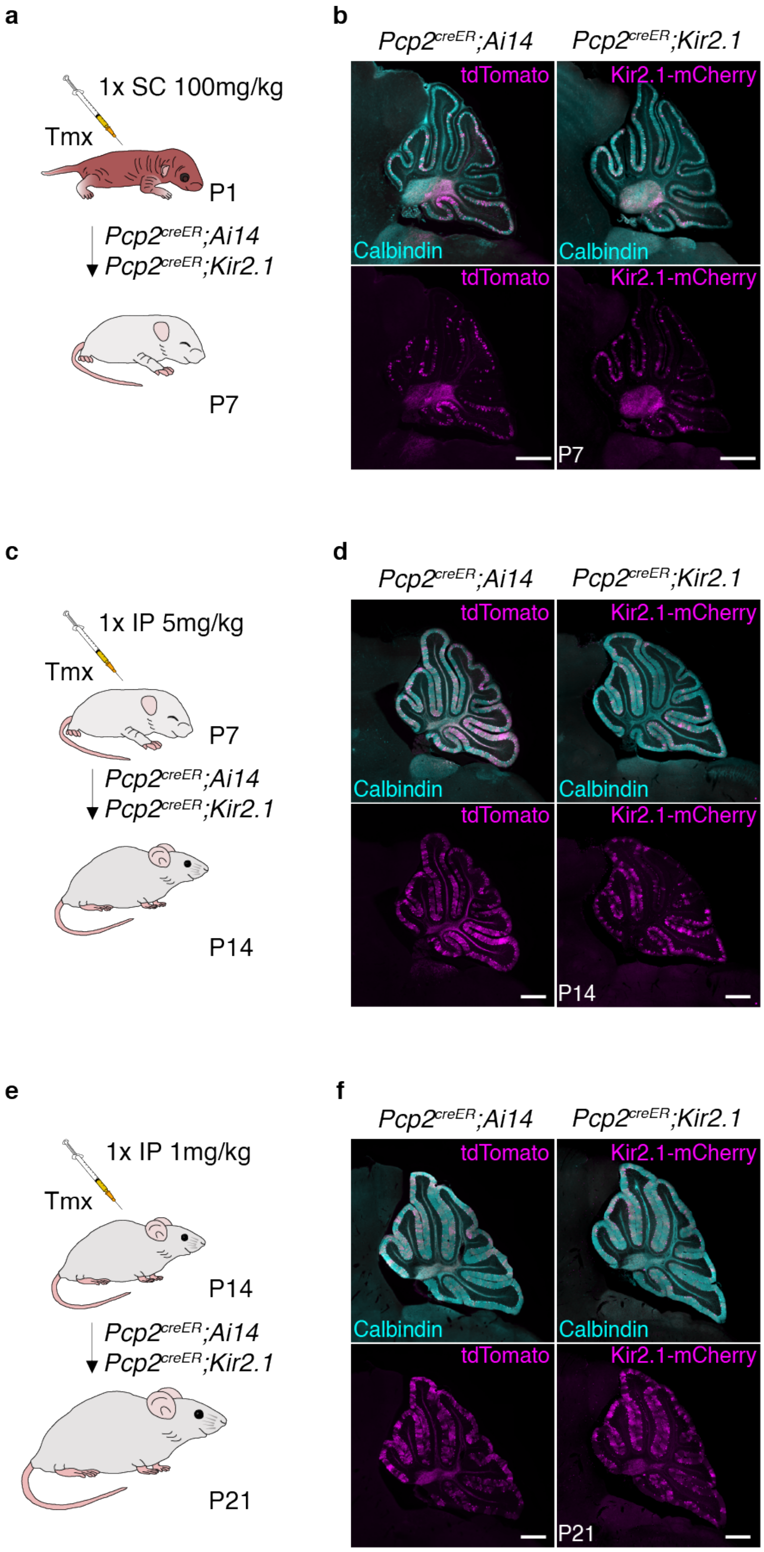
a
1x SC 100mg/kg
Tmx
P1
Pcp2creER;Ai14
Pcp2creER;Kir2.1
P7
b
Pcp2creER;Ai14
Pcp2creER;Kir2.1
tdTomato
Kir2.1-mCherry
Calbindin
Calbindin
tdTomato
Kir2.1-mCherry
P7
c
1x IP 5mg/kg
Tmx
P7
Pcp2creER;Ai14
Pcp2creER;Kir2.1
P14
d
Pcp2creER;Ai14
Pcp2creER;Kir2.1
tdTomato
Kir2.1-mCherry
Calbindin
Calbindin
tdTomato
Kir2.1-mCherry
P14
e
1x IP 1mg/kg
Tmx
P14
Pcp2creER;Ai14
Pcp2creER;Kir2.1
P21
f
Pcp2creER;Ai14
Pcp2creER;Kir2.1
tdTomato
Kir2.1-mCherry
Calbindin
Calbindin
tdTomato
Kir2.1-mCherry
P21

Supplementary Fig. 7 Sparse labeling of Purkinje cells at P7, P14, or P21 in different mouse models. **a** Experimental design. *Pcp2^{creER};Ai14* and *Pcp2^{creER};Kir2.1* pups were injected subcutaneously (SC) with tamoxifen (Tmx, 100mg/kg) at postnatal day 1 (P1), and brains were collected at P7. **b** Sagittal cerebellar sections from *Pcp2^{creER};Ai14 and Pcp2^{creER};Kir2.1* mice at P7 show sparse expression of tdTomato (magenta) or Kir2.1-mCherry (magenta), respectively, in Purkinje cells co-labeled with calbindin (cyan). **c** Experimental design. *Pcp2^{creER};Ai14* and *Pcp2^{creER};Kir2.1* pups were injected intraperitoneally (IP) with Tmx (5mg/kg) at P7, and brains were collected at P14. **d** Sagittal cerebellar sections from *Pcp2^{creER};Ai14 and Pcp2^{creER};Kir2.1* mice at P7 show sparse expression of tdTomato (magenta) or Kir2.1-mCherry (magenta), respectively, in Purkinje cells co-labeled with calbindin (cyan). **e** Experimental design. *Pcp2^{creER};Ai14* and *Pcp2^{creER};Kir2.1* pups were injected IP with Tmx (1mg/kg) at P14, and brains were collected at P21. **f** Sagittal cerebellar sections from *Pcp2^{creER};Ai14 and Pcp2^{creER};Kir2.1* mice at P21 show sparse expression of tdTomato (magenta) or Kir2.1-mCherry (magenta), respectively, in Purkinje cells co-labeled with calbindin (cyan). Scale bars, 500 μm (**b**, **d**, **f**). Data related to Fig. 4.

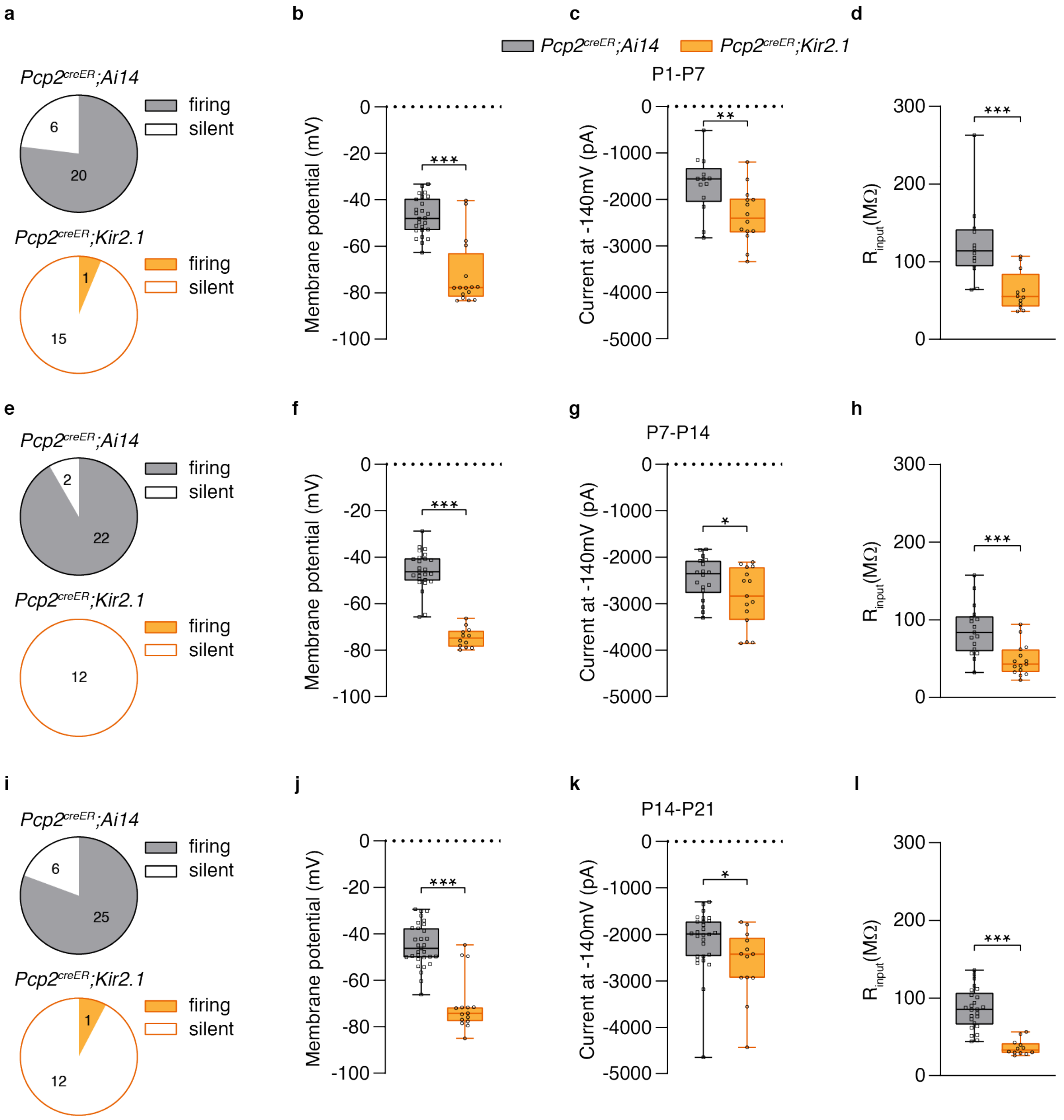

a
b
c
d
Pcp2creER;Ai14
Pcp2creER;Kir2.1
P1-P7
firing
silent
6
20
1
15
Membrane potential (mV)
Current at -140mV (pA)
R input (MΩ)
***
**
e
f
g
h
P7-P14
2
22
12
*
i
j
k
l
P14-P21
6
25
1
12

Supplementary Fig. 8 Overexpression of Kir2.1 decreases the excitability of Purkinje cells at different developmental ages. a Number of firing and silent Purkinje cells in *$Pcp2^{creER}$;Ai14* and *$Pcp2^{creER}$;Kir2.1* mice at postnatal day 7 (P7). Quantification of **b** resting membrane potential in *$Pcp2^{creER}$;Ai14* (n = 26 cells/3 mice) and *$Pcp2^{creER}$;Kir2.1* (n = 16 cells/4 mice), **c** current amplitude at -140mV in *$Pcp2^{creER}$;Ai14* (n = 13 cells/3 mice) and *$Pcp2^{creER}$;Kir2.1* (n = 14 cells/4 mice) and **d** input resistance in *$Pcp2^{creER}$;Ai14* (n = 12 cells/3 mice) and *$Pcp2^{creER}$;Kir2.1* (n = 12 cells/4 mice) Purkinje cells at P7. Mann-Whitney *U* test and unpaired Student's *t*-test: $^{**}P < 0.01$, $^{***}P < 0.001$. **e** Number of firing and silent Purkinje cells in *$Pcp2^{creER}$;Ai14* and *$Pcp2^{creER}$;Kir2.1* mice at P14. Quantification of **f** membrane potential in *$Pcp2^{creER}$;Ai14* (n = 25 cells/2 mice) and *$Pcp2^{creER}$;Kir2.1* (n = 12 cells/5 mice), **g** current amplitude at -140mV in *$Pcp2^{creER}$;Ai14* (n = 18 cells/6 mice) and *$Pcp2^{creER}$;Kir2.1* (n = 15 cells/5 mice) and **h** input resistance in *$Pcp2^{creER}$;Ai14* (n = 17 cells/6 mice) and *$Pcp2^{creER}$;Kir2.1* (n = 15 cells/5 mice) Purkinje cells at P14. Unpaired Student's *t*-test with and without Welch's correction: $^{*}P < 0.05$, $^{***}P < 0.001$. **i** Number of firing and silent Purkinje cells in *$Pcp2^{creER}$;Ai14* and *$Pcp2^{creER}$;Kir2.1* mice at P21. Quantification of **j** membrane potential in *$Pcp2^{creER}$;Ai14* (n = 31 cells/3 mice) and *$Pcp2^{creER}$;Kir2.1* (n = 15 cells/5 mice), **k** current amplitude at -140mV in *$Pcp2^{creER}$;Ai14* (n = 28 cells/7 mice) and *$Pcp2^{creER}$;Kir2.1* (n = 13 cells/4 mice) and **l** input resistance in *$Pcp2^{creER}$;Ai14* (n = 25 cells/7 mice) and *$Pcp2^{creER}$;Kir2.1* (n = 12 cells/4 mice) Purkinje cells at P21. Mann-Whitney *U* test and unpaired Student's *t*-test: $^{*}P < 0.05$, $^{***}P < 0.001$. Box plots indicate the median (middle line), 25th and 75th percentiles (box), and 5th and 95th percentiles (whiskers). Statistical details are provided in Supplementary Table 1. Source data are provided as a Source data file. Data related to Fig. 4.

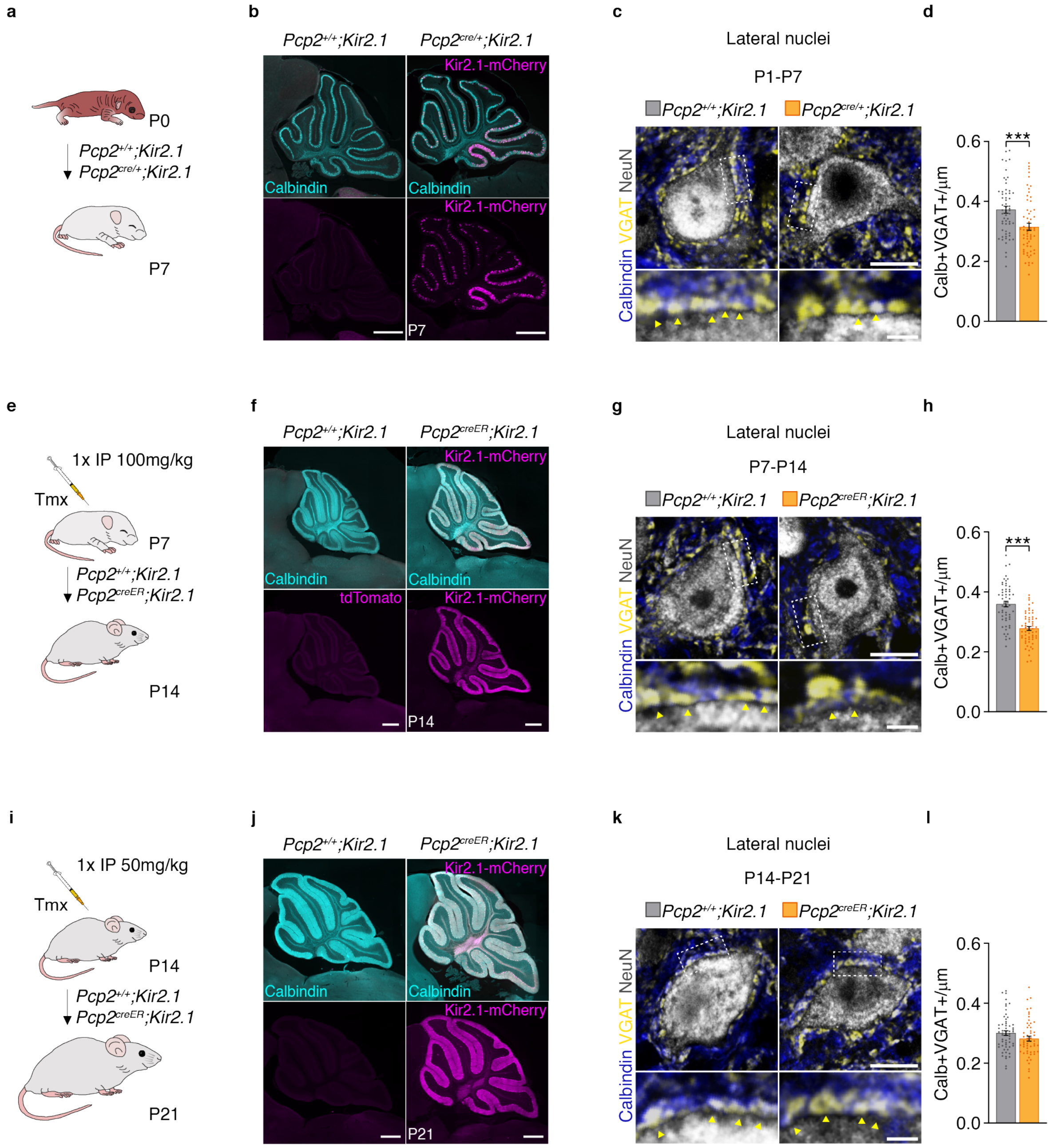

a
P0
Pcp2+/+;Kir2.1
Pcp2cre/+;Kir2.1
P7
b
Pcp2+/+;Kir2.1
Pcp2cre/+;Kir2.1
Kir2.1-mCherry
Calbindin
Calbindin
Kir2.1-mCherry
P7
c
Lateral nuclei
P1-P7
Pcp2+/+;Kir2.1
Pcp2cre/+;Kir2.1
Calbindin VGAT NeuN
d
***
0.6
0.4
0.2
0.0
Calb+VGAT+/µm
e
1x IP 100mg/kg
Tmx
P7
Pcp2+/+;Kir2.1
Pcp2creER;Kir2.1
P14
f
Pcp2+/+;Kir2.1
Pcp2creER;Kir2.1
Kir2.1-mCherry
Calbindin
Calbindin
tdTomato
Kir2.1-mCherry
P14
g
Lateral nuclei
P7-P14
Pcp2+/+;Kir2.1
Pcp2creER;Kir2.1
Calbindin VGAT NeuN
h
***
0.6
0.4
0.2
0.0
Calb+VGAT+/µm
i
1x IP 50mg/kg
Tmx
P14
Pcp2+/+;Kir2.1
Pcp2creER;Kir2.1
P21
j
Pcp2+/+;Kir2.1
Pcp2creER;Kir2.1
Kir2.1-mCherry
Calbindin
Calbindin
Kir2.1-mCherry
P21
k
Lateral nuclei
P14-P21
Pcp2+/+;Kir2.1
Pcp2creER;Kir2.1
Calbindin VGAT NeuN
l
0.6
0.4
0.2
0.0
Calb+VGAT+/µm

Supplementary Fig. 9 Overexpression of Kir2.1 reduces the number of inhibitory Purkinje cell inputs onto lateral cerebellar nuclei neurons during early postnatal development. **a** Experimental design. Brains from conditional *Pcp2*$^{cre/+}$*;Kir2.1* and littermate controls, *Pcp2*$^{+/+}$*;Kir2.1* mice were collected at postnatal day 7 (P7). **b** Sagittal cerebellar sections from *Pcp2*$^{+/+}$*;Kir2.1* and *Pcp2*$^{cre/+}$*;Kir2.1* mice show absence or expression of Kir2.1-mCherry (magenta), respectively, in Purkinje cells labeled with calbindin (cyan). **c** Representative images (top) and high-magnification insets (bottom) showing calbindin-positive (+) (blue) Purkinje cell axon terminals and VGAT+ (yellow) presynaptic boutons surrounding a NeuN+ (grey) neuron in the lateral cerebellar nuclei from *Pcp2*$^{+/+}$*;Kir2.1* and *Pcp2*$^{cre/+}$*;Kir2.1* mice at P7. **d** Density of calbindin+ VGAT+ axon terminals contacting NeuN+ neurons in *Pcp2*$^{+/+}$*;Kir2.1* (n = 58 cells/3 mice) and *Pcp2*$^{cre/+}$*;Kir2.1* (n = 60 cells/3 mice) groups at P7. Mann-Whitney *U* test: *** $P < 0.001$. **e** Experimental design. *Pcp2*$^{+/+}$*;Kir2.1* and *Pcp2*^{creER}*;Kir2.1* pups were injected intraperitoneally (IP) with tamoxifen (Tmx, 100mg/kg) at P7, and brains were collected at P14. **f** Sagittal cerebellar sections from *Pcp2*$^{+/+}$*;Kir2.1 and Pcp2*^{creER}*;Kir2.1* mice at P14 showing absence or expression of Kir2.1-mCherry (magenta), respectively, in Purkinje cells labeled with calbindin (cyan). **g** Representative images (top) and high-magnification insets (bottom) show calbindin+ (blue) Purkinje cell axon terminals and VGAT+ (yellow) presynaptic boutons surrounding a NeuN+ (grey) neuron in the lateral cerebellar nuclei from *Pcp2*$^{+/+}$*;Kir2.1* and *Pcp2*^{creER}*;Kir2.1* mice at P14. **h** Density of calbindin+ VGAT+ axon terminals contacting NeuN+ neurons in *Pcp2*$^{+/+}$*;Kir2.1* (n = 55 cells/3 mice) and *Pcp2*^{creER}*;Kir2.1* (n = 59 cells/3 mice) groups at P14. Unpaired Student's *t*-test with Welch's correction: ***$P < 0.001$. **i** Experimental design. *Pcp2*$^{+/+}$*;Kir2.1* and *Pcp2*^{creER}*;Kir2.1* pups were injected IP with Tmx (50mg/kg) at P14, and brains were collected at P21. **j** Sagittal cerebellar sections from *Pcp2*$^{+/+}$*;Kir2.1* and *Pcp2*^{creER}*;Kir2.1* mice at P21 show absence or expression of Kir2.1-mCherry (magenta), respectively, in Purkinje cells labeled with calbindin (cyan). **k** Representative images (top) and high-magnification insets (bottom) show calbindin+ (blue) Purkinje cell axon terminals and VGAT+ (yellow) presynaptic boutons surrounding a NeuN+ (grey) neuron in the lateral cerebellar nuclei from *Pcp2*$^{+/+}$*;Kir2.1* and *Pcp2*^{creER}*;Kir2.1* mice at P21. **l** Density of calbindin+ VGAT+ axon terminals contacting NeuN+ neurons in *Pcp2*$^{+/+}$*;Kir2.1* (n = 59 cells/3 mice) and *Pcp2*^{creER}*;Kir2.1* (n = 55 cells/3 mice) groups at P21. Unpaired Student's *t*-test. Scale bars, 500 µm (**b**, **f**, **j**); 10 µm (top panels in **c**, **g**, **k**); 2 µm (bottom panels in **c**, **g**, **k**). Data are shown as the mean ± s.e.m. Statistical details are provided in Supplementary Table 1. Source data are provided as a Source data file. Data related to Fig. 4.

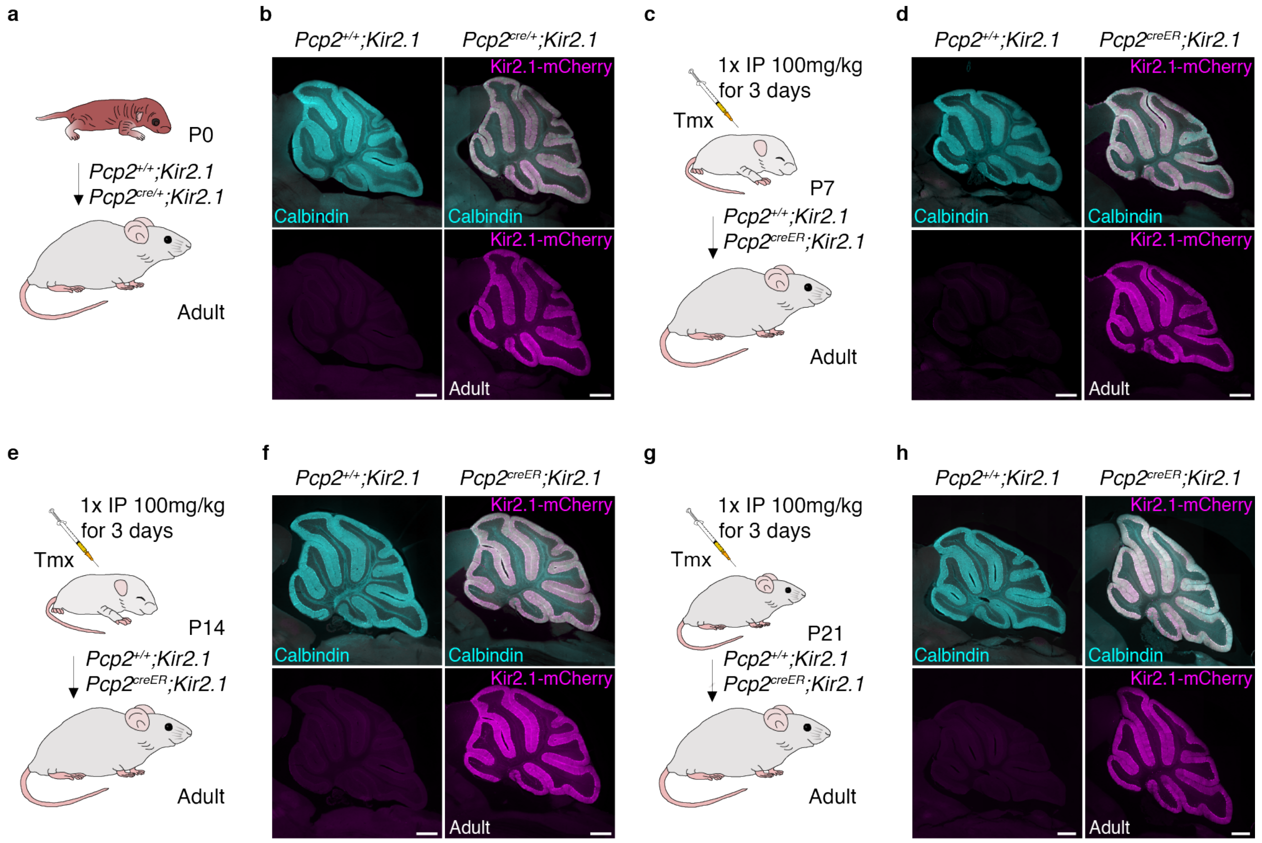

Supplementary Fig. 10 Relative impact of Purkinje cell activity reduction at different developmental stages on balance beam performance in adult mice. a Experimental design. Brains were collected from adult *Pcp2$^{cre/+}$;Kir2.1* mice and littermate controls *Pcp2$^{+/+}$;Kir2.1*. **b** Sagittal cerebellar sections from adult *Pcp2$^{+/+}$;Kir2.1* and *Pcp2$^{cre/+}$;Kir2.1* mice show absence or expression of Kir2.1-mCherry (magenta), respectively, in Purkinje cells labeled with calbindin (cyan). **c** Experimental design. *Pcp2$^{+/+}$;Kir2.1* and *Pcp2^{creER};Kir2.1* pups were injected intraperitoneally (IP) with tamoxifen (Tmx, 100mg/kg) for three consecutive days starting at postnatal day 7 (P7), and brains were collected in adulthood. **d** Sagittal cerebellar sections from adult *Pcp2$^{+/+}$;Kir2.1* and *Pcp2^{creER};Kir2.1* mice show absence or expression of Kir2.1-mCherry (magenta), respectively, in Purkinje cells labeled with calbindin (cyan). **e** Experimental design. *Pcp2$^{+/+}$;Kir2.1* and *Pcp2^{creER};Kir2.1* pups were injected IP with Tmx (100mg/kg) for three consecutive days starting at P14, and brains were collected in adulthood. **f** Sagittal cerebellar sections from adult *Pcp2$^{+/+}$;Kir2.1* and *Pcp2^{creER};Kir2.1* mice show absence or expression of Kir2.1-mCherry (magenta), respectively, in Purkinje cells labeled with calbindin (cyan). **g** Experimental design. *Pcp2$^{+/+}$;Kir2.1* and *Pcp2^{creER};Kir2.1* mice were injected IP with Tmx (100mg/kg) for three consecutive days starting at P21, and brains were collected in adulthood. **h** Sagittal cerebellar sections from adult *Pcp2$^{+/+}$;Kir2.1* and *Pcp2^{creER};Kir2.1* mice show absence or expression of Kir2.1-mCherry (magenta), respectively, in Purkinje cells labeled with calbindin (cyan). Scale bars, 500 μm (**b**, **d**, **f**, **h**). Data related to Fig. 5.

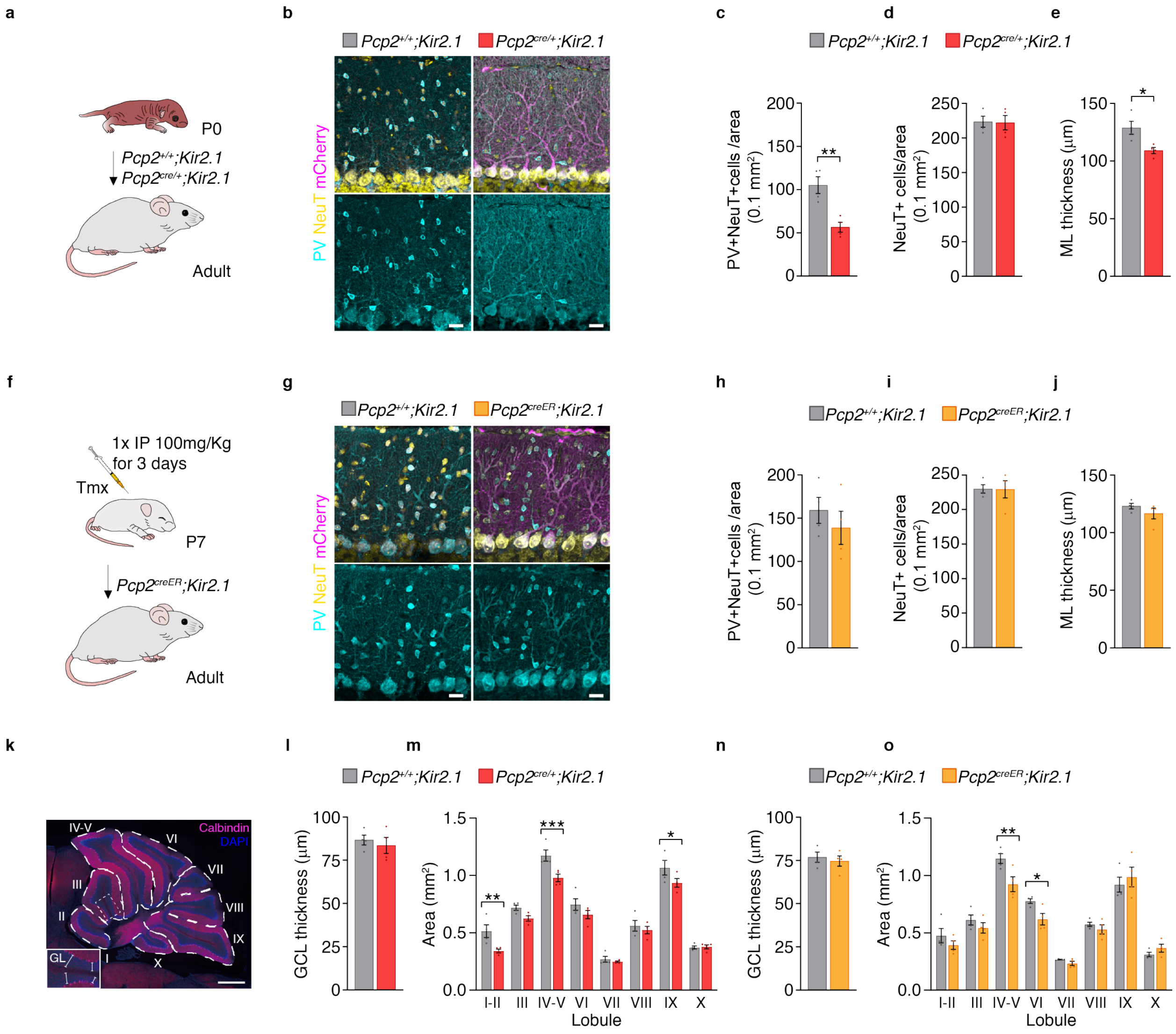

a
b
c
d
e
P0
Pcp2+/+;Kir2.1
Pcp2cre/+;Kir2.1
Adult
PV NeuT mCherry
PV+NeuT+cells /area (0.1 mm2)
NeuT+ cells/area (0.1 mm2)
ML thickness (µm)
f
g
h
i
j
1x IP 100mg/Kg for 3 days
Tmx
P7
Pcp2creER;Kir2.1
Adult
k
l
m
n
o
IV-V
VI
VII
VIII
IX
X
III
II
I
GL
Calbindin
DAPI
GCL thickness (µm)
Area (mm2)
I-II III IV-V VI VII VIII IX X
Lobule

Supplementary Fig. 11 Overexpression of Kir2.1 in Purkinje cells from birth reduces the number of parvalbumin interneurons in adult mice. a Experimental design. Brains were collected from adult *Pcp2*$^{cre/+}$*;Kir2.1* mice and littermate controls *Pcp2*$^{+/+}$*;Kir2.1*. **b** Sagittal cerebellar sections from adult *Pcp2*$^{+/+}$*;Kir2.1* and *Pcp2*$^{cre/+}$*;Kir2.1* mice showing parvalbumin-positive (PV+, cyan) and NeuroTrace-positive (NeuT+; yellow) interneurons without and with Kir2.1-mCherry (magenta) expression, respectively, in Purkinje cells. Quantification of **c** PV+ NeuT+ cells per area of region of interest (ROI); **d** NeuT+ cells per area of ROI; and **e** molecular layer (ML) thickness in *Pcp2*$^{+/+}$*;Kir2.1* (n = 4 mice) and *Pcp2*$^{cre/+}$*;Kir2.1* (n = 4 mice) adults. Unpaired Student's *t*-test: *P* < 0.05, ***P* < 0.01. **f** Experimental design. *Pcp2*$^{+/+}$*;Kir2.1* and *Pcp2*^{creER}*;Kir2.1* pups were injected intraperitoneally (IP) with tamoxifen (100mg/kg) for three consecutive days starting at postnatal day 7 (P7), and brains were collected in adulthood. **g** Sagittal cerebellar sections from adult *Pcp2*$^{+/+}$*;Kir2.1* and *Pcp2*^{creER}*;Kir2.1* mice showing PV+ (cyan) and NeuT+ (yellow) interneurons without and with Kir2.1-mCherry (magenta) expression, respectively, in Purkinje cells. Quantification of **h** PV+ NeuT+ cells per area of ROI; **i** NeuT+ cells per area of ROI; and **j** ML thickness in *Pcp2*$^{+/+}$*;Kir2.1* (n = 4 mice) and *Pcp2*^{creER}*;Kir2.1* (n = 4 mice) adults. Unpaired Student's *t*-test and Mann-Whitney *U* test. **k** Sagittal cerebellar section showing the vermal lobules and the granular cell layer (GCL). Quantification of **l** GCL thickness and **m** lobule area in *Pcp2*$^{+/+}$*;Kir2.1* (n = 4 mice) and *Pcp2*$^{cre/+}$*;Kir2.1* (n = 4 mice) adults. Unpaired Student's *t*-test and two-way ANOVA with multiple comparisons test: **P* < 0.05, ***P* < 0.01, *** *P* < 0.001. Quantification of **n** GCL thickness and **o** lobule area in *Pcp2*$^{+/+}$*;Kir2.1* (n = 4 mice) and *Pcp2*^{creER}*;Kir2.1* (n = 4 mice) adults. Unpaired Student's *t*-test and two-way ANOVA with multiple comparisons test: **P* < 0.05, ***P* < 0.01. Scale bars, 20 µm (**b**, **g**) and 500 µm (**k**). Data are shown as the mean ± s.e.m. Statistical details are provided in Supplementary Table 1. Source data are provided as a Source data file. Data related to Fig. 5.

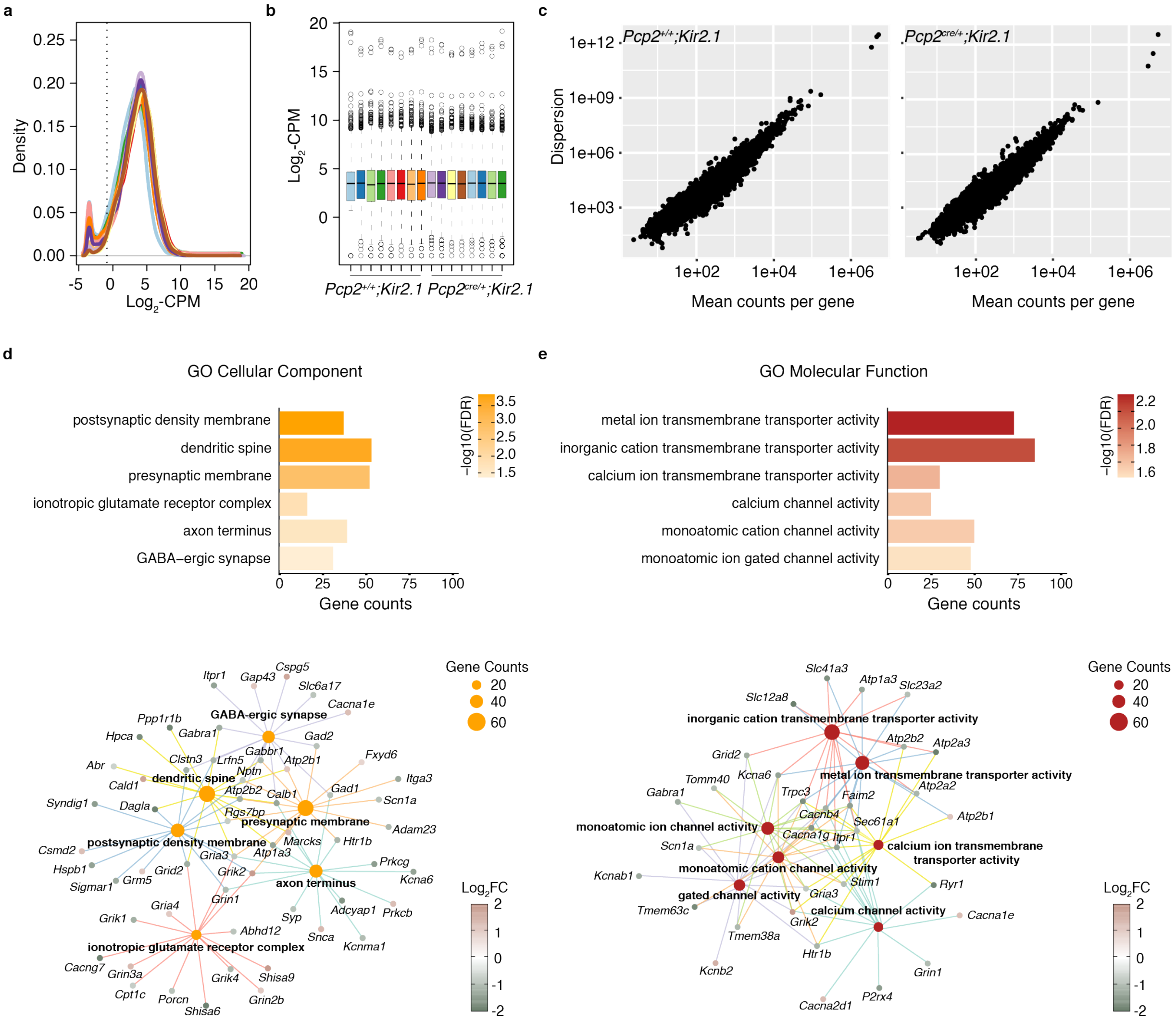

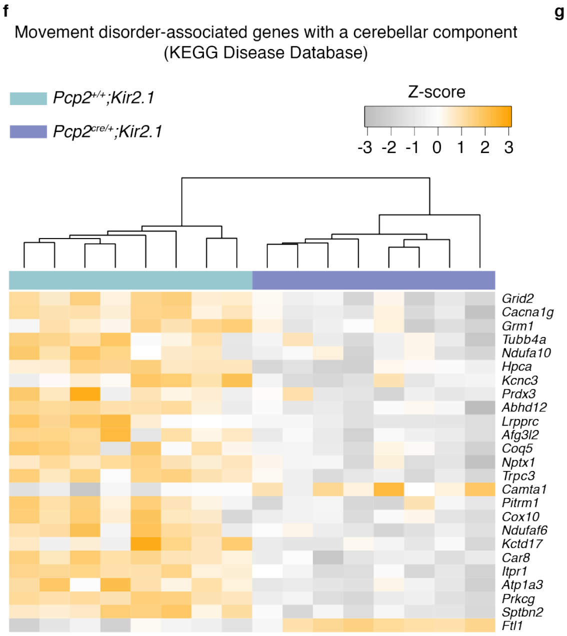

g

Genes	logFC	-log10(FDR)	Disease
Prkcg	-1.54	5.15	Spinocerebellar ataxia
Car8	-1.56	4.03	CAMRQ syndrome
Itpr1	-1.46	3.97	Spinocerebellar ataxia
Trpc3	-1.76	3.76	Spinocerebellar ataxia
Sptbn2	-1.20	3.53	Autosomal dominant and recessive spinocerebellar ataxias
Atp1a3	-1.41	3.27	CAPOS syndrome
Hpca	-1.78	3.25	Primary dystonia
Cacna1g	-1.62	3.18	Spinocerebellar ataxia
Grid2	-1.27	3.08	Autosomal recessive spinocerebellar ataxias
Ftl1	0.93	3.01	Neuroferritinopathy
Nptx1	-1.53	2.89	Spinocerebellar ataxia
Lrpprc	-0.87	2.39	Leigh syndrome
Afg3l2	-1.05	2.17	Spinocerebellar ataxia and Spastic ataxia
Abhd12	-0.93	2.10	PHARC
Grm1	-0.77	1.95	Autosomal dominant and recessive spinocerebellar ataxias
Kcnc3	-1.38	1.95	Spinocerebellar ataxia
Pitrm1	-0.76	1.75	Autosomal recessive spinocerebellar ataxias
Camta1	0.70	1.61	Nonprogressive cerebellar ataxia with intelectual disability
Tubb4a	-0.79	1.58	Pelizaeus-Merzbacher disease and Primary dystonia
Cox10	-0.74	1.57	Leigh syndrome
Ndufaf6	-0.67	1.53	Leigh syndrome
Kctd17	-0.76	1.49	Primary dystonia
Ndufa10	-0.53	1.49	Leigh syndrome
Coq5	-0.71	1.48	Coenzyme Q10 deficiency
Prdx3	-0.74	1.41	Autosomal recessive spinocerebellar ataxias

Supplementary Fig. 12 Validation of RNA sequencing data, gene ontology analysis, and expression of movement disorder-associated genes. **a** Density of log_2-CPM (counts per million) across biological samples (n = 8 mice per group). **b** Boxplots of log_2-CPM values showing mRNA expression distributions for each biological sample. Box plots indicate the median (middle line), 25th and 75th percentiles (box), and 5th and 95th percentiles (whiskers). **c** Gene-count dispersion patterns for *Pcp2*$^{+/+}$*;Kir2.1* and *Pcp2*$^{cre/+}$*;Kir2.1* Purkinje cells at postnatal day 7 (P7). **d**, **e** Selected significantly enriched gene ontology (GO) terms (false discovery rate, FDR ≤ 0.05) and networks based on differentially expressed genes (DEGs) in *Pcp2*$^{cre/+}$*;Kir2.1* Purkinje cells at P7. **d** Cellular Component and **e** Molecular Function GO categories. In the networks, node size reflects the number of enriched genes per GO term, and gene expression level is color-coded (pink, upregulated; green, downregulated; based on log_2 fold change, FC). **f** Heatmap showing *z*-score normalized expression levels of movement disorder-associated genes in *Pcp2*$^{cre/+}$*;Kir2.1* Purkinje cells at P7. Samples and genes were reordered using hierarchical clustering, and the expression level of each gene is color-coded (yellow, high; grey, low). **g** List of 25 disease-associated genes, logFC, -log10(FDR), and associated disorder. CAMRQ, cerebellar ataxia-intellectual disability-disequilibrium syndrome; CAPOS, cerebellar ataxia, areflexia, pes cavus, optic atrophy, and sensorineural hearing loss syndrome; PHARC, polyneuropathy, hearing loss, ataxia, retinitis pigmentosa, and cataract. Data related to Fig. 6.

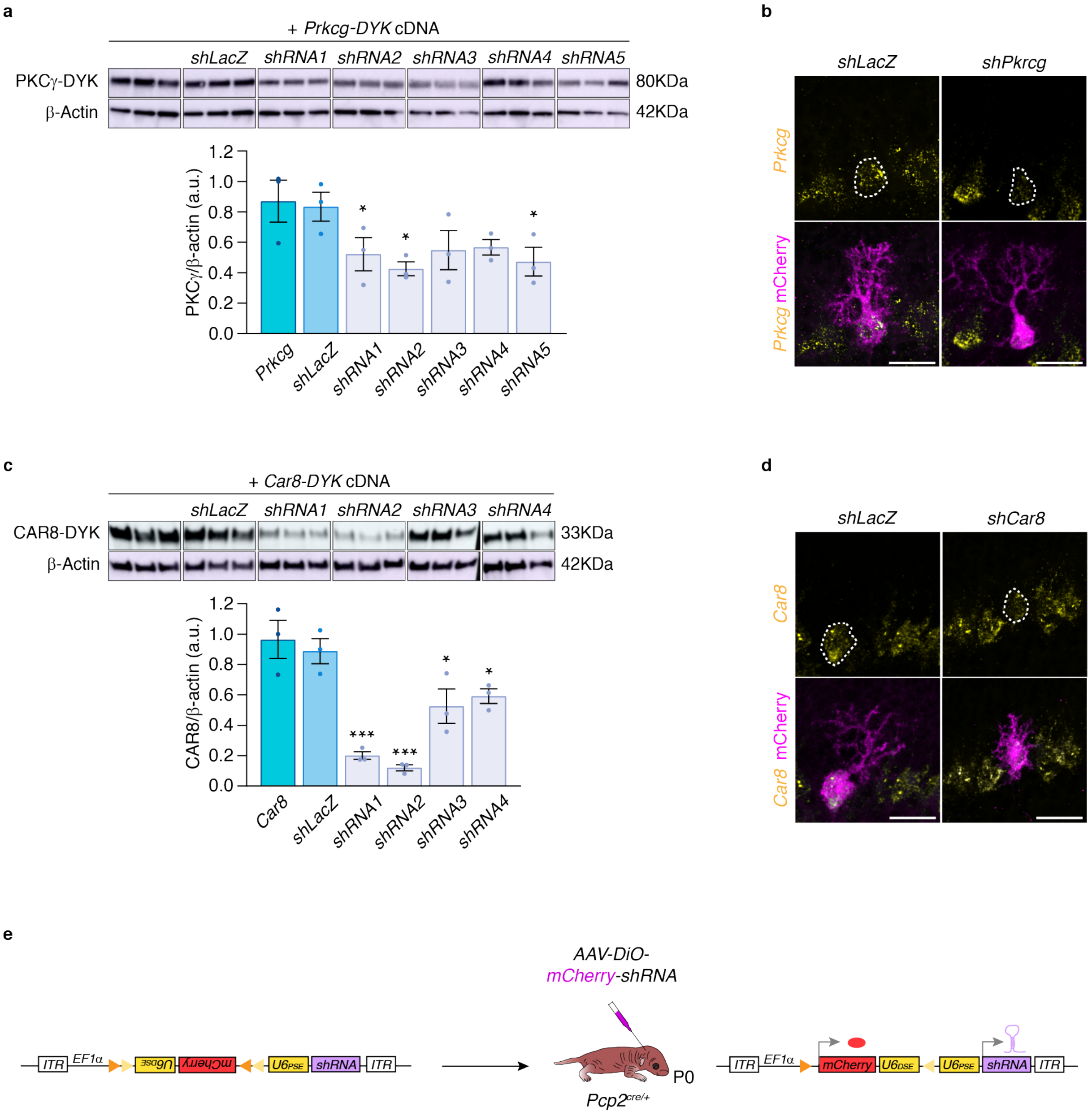

a
+ Prkcg-DYK cDNA
shLacZ
shRNA1
shRNA2
shRNA3
shRNA4
shRNA5
PKCγ-DYK
β-Actin
80KDa
42KDa
PKCγ/β-actin (a.u.)
Prkcg
b
shLacZ
shPkrcg
Prkcg
Prkcg mCherry
c
+ Car8-DYK cDNA
CAR8-DYK
33KDa
CAR8/β-actin (a.u.)
Car8
d
shCar8
Car8 mCherry
e
AAV-DiO-mCherry-shRNA
ITR
EF1α
U6DSE
mCherry
U6PSE
shRNA
P0
Pcp2cre/+

Supplementary Fig. 13 Selection and validation of shRNA sequences targeting *Prkcg* and *Car8* in Purkinje cells. a Western blot analysis and quantification of DYK-tagged PKCγ levels normalized to β-actin from HEK293T cells transfected with *Prkcg-DYK* cDNA alone or co-transfected with either *shLacZ* or one of five *shRNA* constructs targeting *Prkcg* (n = 3). One-way ANOVA with multiple comparisons: * $P < 0.05$. **b** Representative RNAscope images showing expression of *Prkcg* (yellow) and mCherry (magenta) in postnatal day 7 (P7) Purkinje cells from $Pcp2^{cre/+}$ mice injected with adeno-associated virus (AAV) expressing *shLacZ* or *shPrkcg*. **c** Western blot analysis and quantification of DYK-tagged CAR8 levels normalized to β-actin in HEK293T cells transfected with *Car8-DYK* cDNA alone or co-transfected with either *shLacZ* or one of four *shRNA* constructs targeting *Car8* (n = 3). One-way ANOVA with multiple comparisons: * $P < 0.05$, *** $P < 0.001$. **d** Representative RNAscope images showing expression of *Car8* (yellow) and mCherry (magenta) in P7 Purkinje cells from $Pcp2^{cre/+}$ mice injected with AAV expressing *shLacZ* or *shCar8*. Scale bars, 25 µm (**b**, **d**). **e** Schematic illustrates the experimental design and viral construct used to deliver shRNA sequences to Purkinje cells. $Pcp2^{cre/+}$ P0 pups were injected intraventricularly with a viral vector expressing the selected *shRNA* and the reporter gene *mCherry* in a cre-dependent manner. Brains were collected at P7. ITR, inverted terminal repeat; EF1α, elongation factor-1 alpha promoter; $U6_{DSE/PSE}$, U6 promoter distal /proximal sequence elements; shRNA, short hairpin RNA; DiO, double-floxed inverted orientation; Pcp2, Purkinje cell Protein 2 promoter; cre, recombinase. Data are shown as the mean ± s.e.m. Statistical details are provided in Supplementary Table 1. Source data and uncropped blots are provided as a Source data file. Data related to Fig. 7.

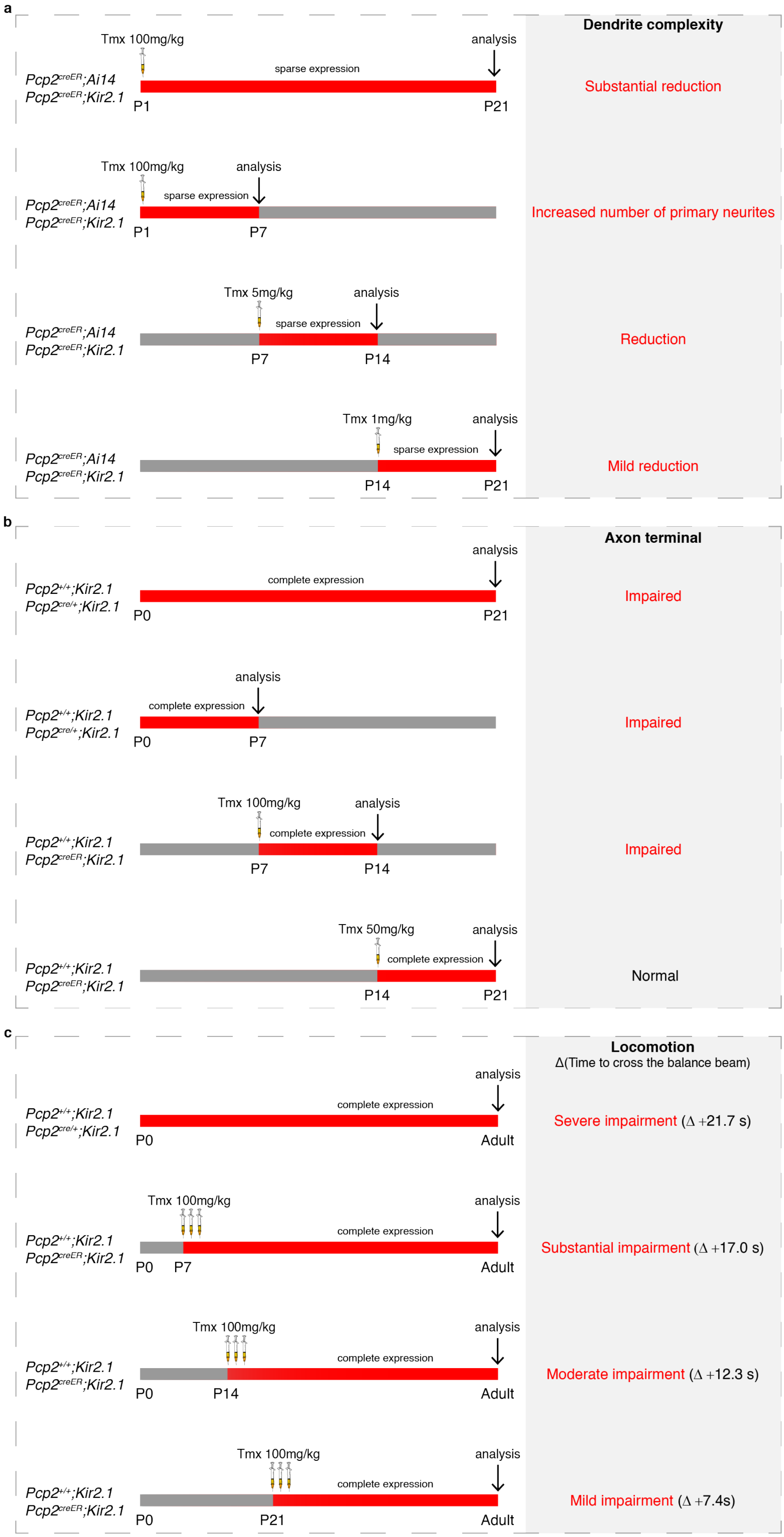
a
Dendrite complexity
Tmx 100mg/kg
analysis
sparse expression
Pcp2creER;Ai14
Pcp2creER;Kir2.1
P1
P21
Substantial reduction
Tmx 100mg/kg
analysis
sparse expression
Pcp2creER;Ai14
Pcp2creER;Kir2.1
P1
P7
Increased number of primary neurites
Tmx 5mg/kg
analysis
sparse expression
Pcp2creER;Ai14
Pcp2creER;Kir2.1
P7
P14
Reduction
Tmx 1mg/kg
analysis
sparse expression
Pcp2creER;Ai14
Pcp2creER;Kir2.1
P14
P21
Mild reduction
b
Axon terminal
analysis
complete expression
Pcp2+/+;Kir2.1
Pcp2cre/+;Kir2.1
P0
P21
Impaired
analysis
complete expression
Pcp2+/+;Kir2.1
Pcp2cre/+;Kir2.1
P0
P7
Impaired
Tmx 100mg/kg
analysis
complete expression
Pcp2+/+;Kir2.1
Pcp2creER;Kir2.1
P7
P14
Impaired
Tmx 50mg/kg
analysis
complete expression
Pcp2+/+;Kir2.1
Pcp2creER;Kir2.1
P14
P21
Normal
c
Locomotion
Δ(Time to cross the balance beam)
analysis
complete expression
Pcp2+/+;Kir2.1
Pcp2cre/+;Kir2.1
P0
Adult
Severe impairment (Δ +21.7 s)
Tmx 100mg/kg
analysis
complete expression
Pcp2+/+;Kir2.1
Pcp2creER;Kir2.1
P0
P7
Adult
Substantial impairment (Δ +17.0 s)
Tmx 100mg/kg
analysis
complete expression
Pcp2+/+;Kir2.1
Pcp2creER;Kir2.1
P0
P14
Adult
Moderate impairment (Δ +12.3 s)
Tmx 100mg/kg
analysis
complete expression
Pcp2+/+;Kir2.1
Pcp2creER;Kir2.1
P0
P21
Adult
Mild impairment (Δ +7.4s)

Supplementary Fig. 14 Summary of experimental designs and resulting phenotypes. Overview of mouse lines, tamoxifen (Tmx) dosage, timing of tamoxifen administration, time of analysis, and observed outcomes for **a** dendrite complexity (related to Figs. 2 and 4), **b** axon terminal density (related to Figs. 2 and 4), and **c** locomotor behavior (related to Figs. 3 and 5). Red shading on the timeline indicates the period of transgene expression (*tdTomato* or *Kir2.1-mCherry*). “sparse” or “complete” expression refers to Purkinje cells labeling. P, postnatal day; Δ, delta-time; s, seconds.

Supplementary Table 1 - Statistical analyses.

Figure	Groups	Test applied	p-value
Fig.1e	Pcp2^{creER};Ai14, Pcp2^{creER};Kir2.1,Ctl and Pcp2^{creER};Kir2.1	two-way repeated measures ANOVA	0.0018
Fig.1h	Pcp2$^{+/+}$;Kir2.1 vs Pcp2$^{cre/+}$;Kir2.1	two-tailed Mann-Whitney U test	<0.0001
Fig.1i	Pcp2$^{+/+}$;Kir2.1 vs Pcp2$^{cre/+}$;Kir2.1	two-tailed Mann-Whitney U test	<0.0001
Fig.1j	Pcp2$^{+/+}$;Kir2.1 vs Pcp2$^{cre/+}$;Kir2.1	two-tailed Mann-Whitney U test	0.0004
Fig.1k	Pcp2$^{+/+}$;Kir2.1 vs Pcp2$^{cre/+}$;Kir2.1		
Supp Fig.2a	Pcp2^{creER};Ai14, Pcp2^{creER};Kir2.1,Ctl, Pcp2^{creER};Kir2.1, Pcp2^{creER};Ai14 + Ba, Pcp2^{creER};Kir2.1,Ctl + Ba and Pcp2^{creER};Kir2.1 + Ba	ordinary one-way ANOVA Tukey's multiple comparisons test post hoc analysis	<0.0001
	Pcp2^{creER};Ai14 vs Pcp2^{creER};Kir2.1,Ctl		0.9893
	Pcp2^{creER};Ai14 vs Pcp2^{creER};Kir2.1		0.0005
	Pcp2^{creER};Kir2.1,Ctl vs Pcp2^{creER};Kir2.1		0.0001
	Pcp2^{creER};Kir2.1 vs Pcp2^{creER};Ai14 + Ba		<0.0001
	Pcp2^{creER};Kir2.1 vs Pcp2^{creER};Kir2.1,Ctl + Ba		<0.0001
	Pcp2^{creER};Kir2.1 vs Pcp2^{creER};Kir2.1 + Ba		<0.0001
Supp Fig.2d	Pcp2^{creER};Ai14, Pcp2^{creER};Kir2.1,Ctl, Pcp2^{creER};Kir2.1, Pcp2^{creER};Ai14 + Ba, Pcp2^{creER};Kir2.1,Ctl + Ba and Pcp2^{creER};Kir2.1 + Ba	ordinary one-way ANOVA Tukey's multiple comparisons test post hoc analysis	0.0001
	Pcp2^{creER};Ai14 vs Pcp2^{creER};Kir2.1,Ctl		>0.9999
	Pcp2^{creER};Ai14 vs Pcp2^{creER};Kir2.1		0.0077
	Pcp2^{creER};Kir2.1,Ctl vs Pcp2^{creER};Kir2.1		0.0061
	Pcp2^{creER};Kir2.1 vs Pcp2^{creER};Ai14 + Ba		0.0319
	Pcp2^{creER};Kir2.1 vs Pcp2^{creER};Kir2.1,Ctl + Ba		0.0001
	Pcp2^{creER};Kir2.1 vs Pcp2^{creER};Kir2.1 + Ba		0.0194
Supp Fig.2e	Pcp2^{creER};Ai14, Pcp2^{creER};Kir2.1,Ctl and Pcp2^{creER};Kir2.1	Kruskal-Wallis with Dunn's multiple comparisons test post hoc analysis	0.0005
	Pcp2^{creER};Ai14 vs Pcp2^{creER};Kir2.1,Ctl		0.6588
	Pcp2^{creER};Ai14 vs Pcp2^{creER};Kir2.1		0.0334
	Pcp2^{creER};Kir2.1,Ctl vs Pcp2^{creER};Kir2.1		0.0004
Fig.2d	Pcp2^{creER};Ai14 vs Pcp2^{creER};Kir2.1 (intersections)	two-tailed unpaired Student's t-test	<0.0001
Fig.2e	Pcp2^{creER};Ai14 vs Pcp2^{creER};Kir2.1 (area)	two-tailed Mann-Whitney U test	<0.0001
Fig.2f	Pcp2^{creER};Ai14 vs Pcp2^{creER};Kir2.1 (longest dendrite)	two-tailed unpaired Student's t-test	<0.0001
Fig.2g	Pcp2^{creER};Ai14 vs Pcp2^{creER};Kir2.1 (ML ratio)	two-tailed Mann-Whitney U test	<0.0001
Fig.2j	Pcp2$^{+/+}$;Kir2.1 vs Pcp2$^{cre/+}$;Kir2.1	two-tailed unpaired Student's t-test	0.0002
Fig.2l	Pcp2$^{+/+}$;Kir2.1 vs Pcp2$^{cre/+}$;Kir2.1	two-tailed unpaired Student's t-test	0.0193
Fig.2m	Pcp2$^{+/+}$;Kir2.1 vs Pcp2$^{cre/+}$;Kir2.1	two-tailed unpaired Student's t-test	0.0110
Supp Fig.3d	Control vs Kir2.1 (intersections)	two-tailed Mann-Whitney U test	<0.0001

Supp Fig.3e	Control vs Kir2.1 (area)	two-tailed Mann-Whitney U test	<0.0001
Supp Fig.3f	Control vs Kir2.1 (longest dendrite)	two-tailed unpaired Student's t-test	<0.0001
Supp Fig.3g	Control vs Kir2.1 (ML ratio)	two-tailed Mann-Whitney U test	<0.0001
Fig.3a	$Pcp2^{+/+}$;Kir2.1 vs $Pcp2^{cre/+}$;Kir2.1 (balance beam – time)	two-tailed Mann-Whitney U test	<0.0001
Fig.3b	$Pcp2^{+/+}$;Kir2.1 vs $Pcp2^{cre/+}$;Kir2.1 (balance beam – number of missteps)	two-tailed Mann-Whitney U test	<0.0001
Fig.3c	$Pcp2^{+/+}$;Kir2.1 vs $Pcp2^{cre/+}$;Kir2.1 (40rpm)	two-way repeated measures ANOVA with Bonferroni's multiple comparisons test post hoc analysis	<0.0001
	$Pcp2^{+/+}$;Kir2.1 vs $Pcp2^{cre/+}$;Kir2.1 (80rpm)		0.0004
Fig.3d	$Pcp2^{+/+}$;Kir2.1 vs $Pcp2^{cre/+}$;Kir2.1 (angle)	two-tailed unpaired Student's t-test	0.0033
Fig.3e	$Pcp2^{+/+}$;Kir2.1 vs $Pcp2^{cre/+}$;Kir2.1 (swing)	two-tailed unpaired Student's t-test	0.0014
Fig.3h	$Pcp2^{+/+}$;Kir2.1 vs $Pcp2^{cre/+}$;Kir2.1 (phase)	mixed-effect analysis repeated measures	<0.0001
Fig.3i	$Pcp2^{+/+}$;Kir2.1 vs $Pcp2^{cre/+}$;Kir2.1 (CR)	two-way repeated measures ANOVA	<0.0001
Supp Fig.4a	$Pcp2^{+/+}$;Kir2.1 vs $Pcp2^{cre/+}$;Kir2.1 (FRHL distance)	two-tailed unpaired Student's t-test	0.4754
	$Pcp2^{+/+}$;Kir2.1 vs $Pcp2^{cre/+}$;Kir2.1 (FRHL duration)	two-tailed unpaired Student's t-test	<0.0001
	$Pcp2^{+/+}$;Kir2.1 vs $Pcp2^{cre/+}$;Kir2.1 (Hind paw distance)	two-tailed unpaired Student's t-test	0.3938
	$Pcp2^{+/+}$;Kir2.1 vs $Pcp2^{cre/+}$;Kir2.1 (Hind paw duration)	two-tailed unpaired Student's t-test	0.0008
Supp Fig.4b	$Pcp2^{+/+}$;Kir2.1 vs $Pcp2^{cre/+}$;Kir2.1 (distance traveled)	two-tailed unpaired Student's t-test with Welch's correction	0.1779
	$Pcp2^{+/+}$;Kir2.1 vs $Pcp2^{cre/+}$;Kir2.1 (speed)	two-tailed unpaired Student's t-test with Welch's correction	0.0374
Supp Fig.4d	$Pcp2^{+/+}$;Kir2.1 vs $Pcp2^{cre/+}$;Kir2.1 (OKR gain)	mixed-effect analysis repeated measures	0.0045
	$Pcp2^{+/+}$;Kir2.1 vs $Pcp2^{cre/+}$;Kir2.1 (OKR phase)	mixed-effect analysis repeated measures	0.0424
Supp Fig.4e	$Pcp2^{+/+}$;Kir2.1 vs $Pcp2^{cre/+}$;Kir2.1 (VOR gain)	mixed-effect analysis repeated measures	0.2137
	$Pcp2^{+/+}$;Kir2.1 vs $Pcp2^{cre/+}$;Kir2.1 (VOR phase)	mixed-effect analysis repeated measures	0.2160
Supp Fig.4f	$Pcp2^{+/+}$;Kir2.1 vs $Pcp2^{cre/+}$;Kir2.1 (VVOR gain)	mixed-effect analysis repeated measures	0.8368
	$Pcp2^{+/+}$;Kir2.1 vs $Pcp2^{cre/+}$;Kir2.1 (VVOR phase)	mixed-effect analysis repeated measures	0.5219
Supp Fig.4g	$Pcp2^{+/+}$;Kir2.1 vs $Pcp2^{cre/+}$;Kir2.1 (gain)	mixed-effect analysis repeated measures	<0.0001
Supp Fig.4h	$Pcp2^{+/+}$;Kir2.1 vs $Pcp2^{cre/+}$;Kir2.1 (CR amplitude)	mixed-effect analysis repeated measures	<0.0001
Supp Fig.4i	$Pcp2^{+/+}$;Kir2.1 vs $Pcp2^{cre/+}$;Kir2.1 (fraction of eyelid closure)	two-way repeated measures ANOVA	<0.0001

Supp Fig.4j	Pcp2$^{+/+}$;Kir2.1 vs Pcp2$^{cre/+}$;Kir2.1 (UR onset	two-tailed Mann-Whitney U test	0.0505
	Pcp2$^{+/+}$;Kir2.1 vs Pcp2$^{cre/+}$;Kir2.1 (UR peak time)	two-tailed unpaired Student's t-test	0.6368
Supp Fig.5c	Pcp2$^{+/+}$;Kir2.1 vs Pcp2$^{cre/+}$;Kir2.1	two-tailed Mann-Whitney U test	<0.0001
Supp Fig.5d	Pcp2$^{+/+}$;Kir2.1 vs Pcp2$^{cre/+}$;Kir2.1	two-tailed unpaired Student's t-test with Welch's correction	<0.0001
Supp Fig.5e	Pcp2$^{+/+}$;Kir2.1 vs Pcp2$^{cre/+}$;Kir2.1	two-tailed unpaired Student's t-test with Welch's correction	0.3600
Supp Fig.5f	Pcp2$^{+/+}$;Kir2.1 vs Pcp2$^{cre/+}$;Kir2.1	two-tailed unpaired Student's t-test	0.5038
Supp Fig.5h	Pcp2$^{+/+}$;Kir2.1 vs Pcp2$^{cre/+}$;Kir2.1	two-tailed unpaired Student's t-test with Welch's correction	0.0166
Supp Fig.5i	Pcp2$^{+/+}$;Kir2.1 vs Pcp2$^{cre/+}$;Kir2.1	two-tailed Mann-Whitney U test	0.0893
Supp Fig.5i	Pcp2$^{+/+}$;Kir2.1 vs Pcp2$^{cre/+}$;Kir2.1	two-tailed Mann-Whitney U test	0.0893
Supp Fig.5i	Pcp2$^{+/+}$;Kir2.1 vs Pcp2$^{cre/+}$;Kir2.1	two-tailed Mann-Whitney U test	0.0893
Supp Fig.5i	Pcp2$^{+/+}$;Kir2.1 vs Pcp2$^{cre/+}$;Kir2.1	two-tailed Mann-Whitney U test	0.0893
Supp Fig.5i	Pcp2$^{+/+}$;Kir2.1 vs Pcp2$^{cre/+}$;Kir2.1	two-tailed Mann-Whitney U test	0.0893
Supp Fig.5i	Pcp2$^{+/+}$;Kir2.1 vs Pcp2$^{cre/+}$;Kir2.1	two-tailed Mann-Whitney U test	0.0893
Supp Fig.6c	Pcp2$^{+/+}$;Kir2.1 vs Pcp2$^{cre/+}$;Kir2.1	two-tailed Mann-Whitney U test	0.0286
Supp Fig.6d	Pcp2$^{+/+}$;Kir2.1 vs Pcp2$^{cre/+}$;Kir2.1	two-tailed unpaired Student's t-test	0.0080
Supp Fig.6e	Pcp2$^{+/+}$;Kir2.1 vs Pcp2$^{cre/+}$;Kir2.1	two-tailed Mann-Whitney U test	0.0286
Supp Fig.6g	Pcp2$^{+/+}$;Kir2.1 vs Pcp2$^{cre/+}$;Kir2.1	two-tailed unpaired Student's t-test	0.0097
Supp Fig.6h	Pcp2$^{+/+}$;Kir2.1 vs Pcp2$^{cre/+}$;Kir2.1	two-tailed unpaired Student's t-test	0.0093
Fig.4d	Pcp2^{creER};Ai14 vs Pcp2^{creER};Kir2.1 (intersections)	two-tailed Mann-Whitney U test	0.0455
	Pcp2^{creER};Ai14 vs Pcp2^{creER};Kir2.1 (area)	two-tailed Mann-Whitney U test	0.8336
	Pcp2^{creER};Ai14 vs Pcp2^{creER};Kir2.1 (longest dendrite)	two-tailed Mann-Whitney U test	0.0992
	Pcp2^{creER};Ai14 vs Pcp2^{creER};Kir2.1 (neurites)	two-tailed Mann-Whitney U test	<0.0001
Fig.4h	Pcp2^{creER};Ai14 vs Pcp2^{creER};Kir2.1 (intersections)	two-tailed Mann-Whitney U test	<0.0001
	Pcp2^{creER};Ai14 vs Pcp2^{creER};Kir2.1 (area)	two-tailed Mann-Whitney U test	<0.0001
	Pcp2^{creER};Ai14 vs Pcp2^{creER};Kir2.1 (longest dendrite)	two-tailed unpaired Student's t-test	<0.0001
	Pcp2^{creER};Ai14 vs Pcp2^{creER};Kir2.1 (ML ratio)	two-tailed Mann-Whitney U test	<0.0001
Fig.4l	Pcp2^{creER};Ai14 vs Pcp2^{creER};Kir2.1 (intersections)	two-tailed Mann-Whitney U test	0.0110

	$Pcp2^{creER}$;Ai14 vs $Pcp2^{creER}$;Kir2.1 (area)	two-tailed unpaired Student's t-test	<0.0001
	$Pcp2^{creER}$;Ai14 vs $Pcp2^{creER}$;Kir2.1 (longest dendrite)	two-tailed Mann-Whitney U test	<0.0001
	$Pcp2^{creER}$;Ai14 vs $Pcp2^{creER}$;Kir2.1 (ML ratio)	two-tailed Mann-Whitney U test	0.0638
Supp Fig.8b	$Pcp2^{creER}$;Ai14 vs $Pcp2^{creER}$;Kir2.1 (P1-P7) – (membrane potential)	two-tailed Mann-Whitney U test	<0.0001
Supp Fig.8c	$Pcp2^{creER}$;Ai14 vs $Pcp2^{creER}$;Kir2.1 (P1-P7) – (current at -140 mV)	two-tailed unpaired Student's t-test	0.0093
Supp Fig.8d	$Pcp2^{creER}$;Ai14 vs $Pcp2^{creER}$;Kir2.1 (P1-P7) – (input resistance)	two-tailed Mann-Whitney U test	0.0001
Supp Fig.8f	$Pcp2^{creER}$;Ai14 vs $Pcp2^{creER}$;Kir2.1 (P7-P14) – (membrane potential)	two-tailed unpaired Student's t-test with Welch's correction	<0.0001
Supp Fig.8g	$Pcp2^{creER}$;Ai14 vs $Pcp2^{creER}$;Kir2.1 (P7-P14) – (current at -140 mV)	two-tailed unpaired Student's t-test	0.0424
Supp Fig.8h	$Pcp2^{creER}$;Ai14 vs $Pcp2^{creER}$;Kir2.1 (P7-P14) – (input resistance)	two-tailed unpaired Student's t-test	0.0004
Supp Fig.8j	$Pcp2^{creER}$;Ai14 vs $Pcp2^{creER}$;Kir2.1 (P14-P21) – (membrane potential)	two-tailed Mann-Whitney U test	<0.0001
Supp Fig.8k	$Pcp2^{creER}$;Ai14 vs $Pcp2^{creER}$;Kir2.1 (P14-P21) – (current at -140 mV)	two-tailed Mann-Whitney U test	0.0261
Supp Fig.8l	$Pcp2^{creER}$;Ai14 vs $Pcp2^{creER}$;Kir2.1 (P7-P14) – (input resistance)	two-tailed Mann-Whitney U test	<0.0001
Supp Fig.9d	$Pcp2^{+/+}$;Kir2.1 vs $Pcp2^{cre/+}$;Kir2.1	two-tailed Mann-Whitney U test	0.0007
Supp Fig.9h	$Pcp2^{+/+}$;Kir2.1 vs $Pcp2^{creER}$;Kir2.1	two-tailed unpaired Student's t-test with Welch's correction	<0.0001
Supp Fig.9l	$Pcp2^{+/+}$;Kir2.1 vs $Pcp2^{creER}$;Kir2.1	two-tailed unpaired Student's t-test	0.1087
Fig.5b	$Pcp2^{+/+}$;Kir2.1, $Pcp2^{cre/+}$;Kir2.1 and $Pcp2^{creER}$;Kir2.1	mixed-effect analysis repeated measures Tukey's multiple comparisons test post hoc analysis	0,0011
	$Pcp2^{+/+}$;Kir2.1 vs $Pcp2^{creER}$;Kir2.1 (P7-Adult)		<0,0001
	$Pcp2^{+/+}$;Kir2.1 vs $Pcp2^{creER}$;Kir2.1 (P14-Adult)		<0,0001
	$Pcp2^{+/+}$;Kir2.1 vs $Pcp2^{creER}$;Kir2.1 (P21-Adult)		0,0039
	$Pcp2^{cre/+}$;Kir2.1 (P0) vs $Pcp2^{creER}$;Kir2.1 (P7)		0,0552
	$Pcp2^{cre/+}$;Kir2.1 (P0) vs $Pcp2^{creER}$;Kir2.1 (P14)		0,0053
	$Pcp2^{cre/+}$;Kir2.1 (P0) vs $Pcp2^{creER}$;Kir2.1 (P21)		<0,0001
	$Pcp2^{creER}$;Kir2.1 (P7) vs $Pcp2^{creER}$;Kir2.1 (P14)		0,8303
	$Pcp2^{creER}$;Kir2.1 (P7) vs $Pcp2^{creER}$;Kir2.1 (P21)		0,0039
	$Pcp2^{creER}$;Kir2.1 (P14) vs $Pcp2^{creER}$;Kir2.1 (P21)		0,0458
Fig.5c	$Pcp2^{+/+}$;Kir2.1, $Pcp2^{cre/+}$;Kir2.1 and $Pcp2^{creER}$;Kir2.1	mixed-effect analysis repeated measures Tukey's multiple	<0,0001

		comparisons test post hoc analysis	
	$Pcp2^{+/+}$;Kir2.1 vs $Pcp2^{creER}$;Kir2.1 (P7-Adult)		<0,0001
	$Pcp2^{+/+}$;Kir2.1 vs $Pcp2^{creER}$;Kir2.1 (P14-Adult)		<0,0001
	$Pcp2^{+/+}$;Kir2.1 vs $Pcp2^{creER}$;Kir2.1 (P21-Adult)		<0,0001
	$Pcp2^{cre/+}$;Kir2.1 (P0) vs $Pcp2^{creER}$;Kir2.1 (P7)		0,0139
	$Pcp2^{cre/+}$;Kir2.1 (P0) vs $Pcp2^{creER}$;Kir2.1 (P14)		<0,0001
	$Pcp2^{cre/+}$;Kir2.1 (P0) vs $Pcp2^{creER}$;Kir2.1 (P21)		<0,0001
	$Pcp2^{creER}$;Kir2.1 (P7) vs $Pcp2^{creER}$;Kir2.1 (P14)		<0,0001
	$Pcp2^{creER}$;Kir2.1 (P7) vs $Pcp2^{creER}$;Kir2.1 (P21)		0,0009
	$Pcp2^{creER}$;Kir2.1 (P14) vs $Pcp2^{creER}$;Kir2.1 (P21)		0,1720
Supp Fig.11c	$Pcp2^{+/+}$;Kir2.1 vs $Pcp2^{cre/+}$;Kir2.1	two-tailed unpaired Student's t-test	0.0048
Supp Fig.11d	$Pcp2^{+/+}$;Kir2.1 vs $Pcp2^{cre/+}$;Kir2.1	two-tailed unpaired Student's t-test	0.9192
Supp Fig.11e	$Pcp2^{+/+}$;Kir2.1 vs $Pcp2^{cre/+}$;Kir2.1	two-tailed unpaired Student's t-test	0.0200
Supp Fig.11h	$Pcp2^{+/+}$;Kir2.1 vs $Pcp2^{creER}$;Kir2.1	two-tailed unpaired Student's t-test	0.4371
Supp Fig.11i	$Pcp2^{+/+}$;Kir2.1 vs $Pcp2^{creER}$;Kir2.1	two-tailed unpaired Student's t-test	0.9728
Supp Fig.11j	$Pcp2^{+/+}$;Kir2.1 vs $Pcp2^{creER}$;Kir2.1	two-tailed Mann-Whitney U test	0.2000
Supp Fig.11l	$Pcp2^{+/+}$;Kir2.1 vs $Pcp2^{cre/+}$;Kir2.1	two-tailed unpaired Student's t-test	0.5876
Supp Fig.11m	$Pcp2^{+/+}$;Kir2.1 vs $Pcp2^{cre/+}$;Kir2.1	two-way ANOVA with multiple comparisons test	<0.0001
	I-II		0.0018
	III		0.0747
	IV-V		0.0005
	VI		0.0968
	VII		0.6869
	VIII		0.4772
	IX		0.0144
	IX		0.9153
Supp Fig.11n	$Pcp2^{+/+}$;Kir2.1 vs $Pcp2^{creER}$;Kir2.1	two-tailed unpaired Student's t-test	0.6075
Supp Fig.11o	$Pcp2^{+/+}$;Kir2.1 vs $Pcp2^{creER}$;Kir2.1	two-way ANOVA with multiple comparisons test	0.0161
	I-II		0.2115
	III		0.2959
	IV-V		0.0013
	VI		0.0427
	VII		0.6799
	VIII		0.5067

	IX		0.3094
	IX		0.4105
Fig.7d	shLacZ, shPrkcg and shCar8 (intersections)	Kruskal-Wallis with Dunn’s multiple comparisons test post hoc analysis	<0.0001
	shLacZ vs. shPKC		<0.0001
	shLacZ vs. shCar8		>0.9999
Fig.7e	shLacZ, shPrkcg and shCar8 (area)	Kruskal-Wallis with Dunn’s multiple comparisons test post hoc analysis	<0.0001
	shLacZ vs. shPKC		<0.0001
	shLacZ vs. shCar8		0.5769
Fig.7f	shLacZ, shPrkcg and shCar8 (longest dendrite)	Kruskal-Wallis with Dunn’s multiple comparisons test post hoc analysis	<0.0001
	shLacZ vs. shPKC		<0.0001
	shLacZ vs. shCar8		0.0346
Fig.7g	shLacZ, shPrkcg and shCar8 (neurites)	Kruskal-Wallis with Dunn’s multiple comparisons test post hoc analysis	<0.0001
	shLacZ vs. shPKC		0.0011
	shLacZ vs. shCar8		<0.0001
Supp Fig.13a	Prkcg, shLacZ, shRNA1, shRNA2, shRNA3, shRNA4 and shRNA5	ordinary one-way ANOVA multiple comparisons	0.0396
	Lacz vs. PKC		0.8011
	Lacz vs. shRNA1		0.0445
	Lacz vs. shRNA2		0.0120
	Lacz vs. shRNA3		0.0635
	Lacz vs. shRNA4		0.0810
	Lacz vs. shRNA5		0.0229
Supp Fig.13c	Car8, shLacZ, shRNA1, shRNA2, shRNA3 and shRNA4	ordinary one-way ANOVA multiple comparisons	<0.0001
	Lacz vs. Car8		0.9879
	Lacz vs. shRNA1		<0.0001
	Lacz vs. shRNA2		<0.0001
	Lacz vs. shRNA3		0.0145
	Lacz vs. shRNA4		0.0112

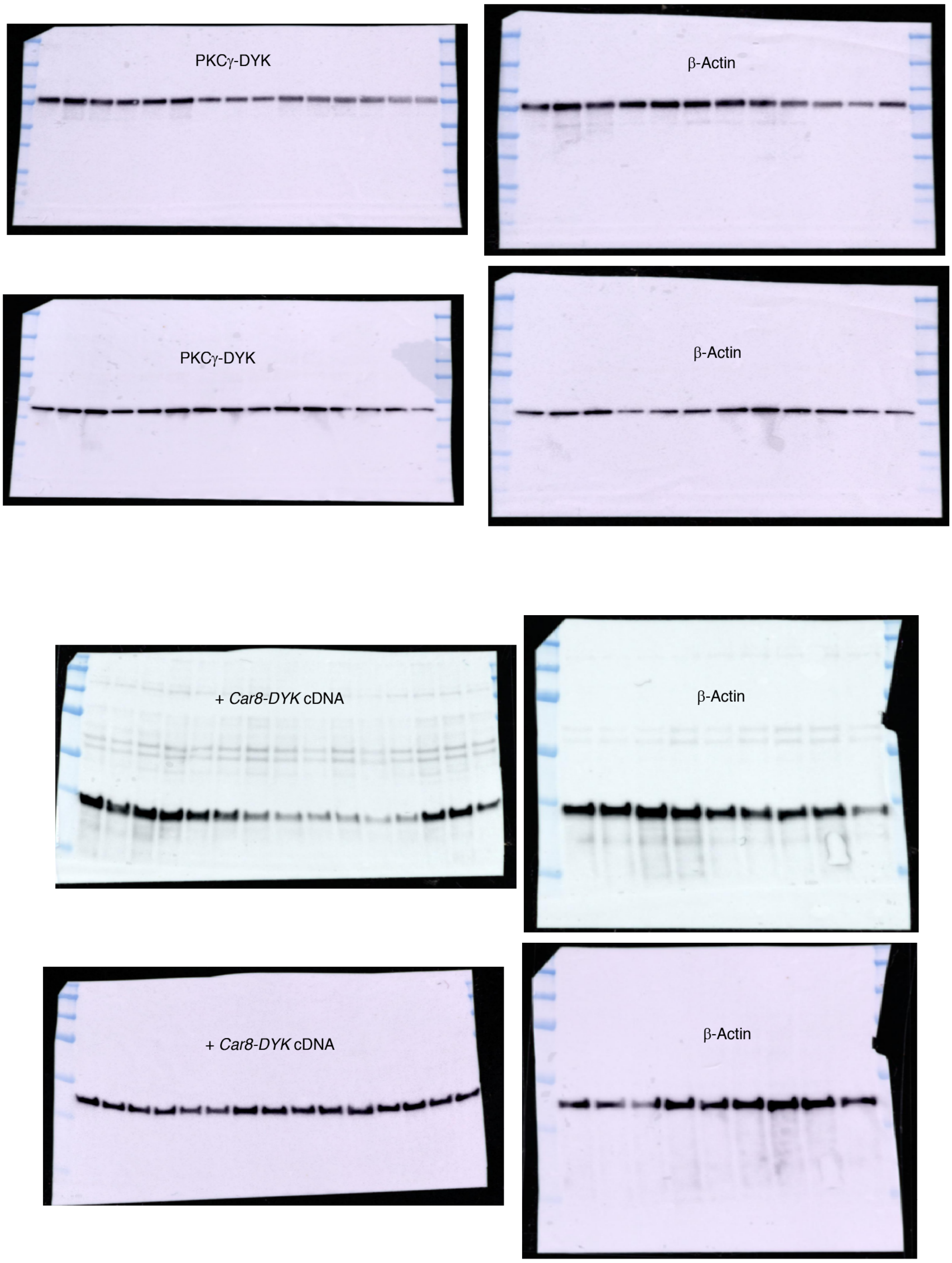

Uncropped blots.
