# Supplementary material for: Purkinje cell intrinsic activity shapes cerebellar development and function
Source: Nat Commun. 2026 Mar 9;17:3688. doi: 10.1038/s41467-026-70355-w (PMC13100008; doi:10.1038/s41467-026-70355-w)
Supplement: Supplementary file 2 — Description of Additional Supplementary Files [file 41467_2026_70355_MOESM2_ESM.pdf]

Description of Additional Supplementary Files

File name: Supplementary Movie 1

Description: Adult *Pcp2*$^{+/+}$*;Kir2.1* mouse successfully performs the 12 mm flat beam task.

File name: Supplementary Movie 2

Description: Adult *Pcp2*$^{cre/+}$*;Kir2.1* mouse shows impaired motor coordination in the 12 mm flat beam task.

File name: Supplementary Movie 3

Description: Adult *Pcp2*$^{cre/+}$*;Kir2.1* mouse shows impaired performance on the accelerating rotarod compared with *Pcp2*$^{+/+}$*;Kir2.1* controls.

File name: Supplementary Movie 4

Description: Adult *Pcp2*$^{+/+}$*;Kir2.1* mouse successfully performs the LocoMouse test.

File name: Supplementary Movie 5

Description: Adult *Pcp2*$^{cre/+}$*;Kir2.1* mouse shows impaired interlimb coordination in the LocoMouse test.
