# Supplementary material for: Purkinje cell intrinsic activity shapes cerebellar development and function
Source: Nat Commun. 2026 Mar 9;17:3688. doi: 10.1038/s41467-026-70355-w (PMC13100008; doi:10.1038/s41467-026-70355-w)
Supplement: Supplementary file 8 — Reporting Summary [file 41467_2026_70355_MOESM8_ESM.pdf]

nature portfolio

Corresponding author(s): Catarina Osório; Martijn Schonewille

Last updated by author(s): Feb 3, 2026

Reporting Summary

Nature Portfolio wishes to improve the reproducibility of the work that we publish. This form provides structure for consistency and transparency in reporting. For further information on Nature Portfolio policies, see our Editorial Policies and the Editorial Policy Checklist.

Statistics

For all statistical analyses, confirm that the following items are present in the figure legend, table legend, main text, or Methods section.

n/a	Confirmed	
☐	☒	The exact sample size (*n*) for each experimental group/condition, given as a discrete number and unit of measurement
☐	☒	A statement on whether measurements were taken from distinct samples or whether the same sample was measured repeatedly
☐	☒	The statistical test(s) used AND whether they are one- or two-sided<br>*Only common tests should be described solely by name; describe more complex techniques in the Methods section.*
☐	☒	A description of all covariates tested
☐	☒	A description of any assumptions or corrections, such as tests of normality and adjustment for multiple comparisons
☐	☒	A full description of the statistical parameters including central tendency (e.g. means) or other basic estimates (e.g. regression coefficient) AND variation (e.g. standard deviation) or associated estimates of uncertainty (e.g. confidence intervals)
☐	☒	For null hypothesis testing, the test statistic (e.g. *F*, *t*, *r*) with confidence intervals, effect sizes, degrees of freedom and *P* value noted<br>*Give P values as exact values whenever suitable.*
☒	☐	For Bayesian analysis, information on the choice of priors and Markov chain Monte Carlo settings
☒	☐	For hierarchical and complex designs, identification of the appropriate level for tests and full reporting of outcomes
☒	☐	Estimates of effect sizes (e.g. Cohen's *d*, Pearson's *r*), indicating how they were calculated

Our web collection on statistics for biologists contains articles on many of the points above.

Software and code

Policy information about availability of computer code

Data collection	Commercial softwares for collection of electrophysiological data: Spike2 (CED), Patchmaster (HEKA), Spiketrain (Neurasmus BV) and Imaging: Zen (Zeiss).
Data analysis	This study did not generate original codes. For LocoMouse analyses, custom-written Python (v3.7) code previously reported has been deposited at https://github.com/BaduraLab/DLC_analysis. For Compensatory eye movement analyses, code previously reported has been deposited at https://github.com/MSchonewille/iMove. For Eyeblink conditioning analyses, custom-written MATLAB R2018a code has been deposited at https://github.com/francescafiocchi91/Eyeblink_Conditioning.

For manuscripts utilizing custom algorithms or software that are central to the research but not yet described in published literature, software must be made available to editors and reviewers. We strongly encourage code deposition in a community repository (e.g. GitHub). See the Nature Portfolio guidelines for submitting code & software for further information.

Data

Policy information about availability of data

All manuscripts must include a data availability statement. This statement should provide the following information, where applicable:

- Accession codes, unique identifiers, or web links for publicly available datasets
- A description of any restrictions on data availability
- For clinical datasets or third party data, please ensure that the statement adheres to our policy

Sequencing data generated in this study have been deposited in the National Center for Biotechnology Information (NCBI) Gene Expression Omnibus (GEO) under

the accession number GSE294208 and are publicly available. Source data are provided with this paper.

Research involving human participants, their data, or biological material

Policy information about studies with human participants or human data. See also policy information about sex, gender (identity/presentation), and sexual orientation and race, ethnicity and racism.

Reporting on sex and gender	n/a
Reporting on race, ethnicity, or other socially relevant groupings	n/a
Population characteristics	n/a
Recruitment	n/a
Ethics oversight	n/a

Note that full information on the approval of the study protocol must also be provided in the manuscript.

Field-specific reporting

Please select the one below that is the best fit for your research. If you are not sure, read the appropriate sections before making your selection.

[x] Life sciences [ ] Behavioural & social sciences [ ] Ecological, evolutionary & environmental sciences

For a reference copy of the document with all sections, see nature.com/documents/nr-reporting-summary-flat.pdf

Life sciences study design

All studies must disclose on these points even when the disclosure is negative.

Sample size	Sample sizes were not pre-determined. Sample sizes are in line with previous literature using similar procedures.
Data exclusions	For ex vivo physiology, data were excluded if the input resistance exceeded 25 MΩ. For eye movement experiments, data were excluded if the goodness-of-fit was below 0.2.
Replication	For immunohistological data, all experiments were replicated in at least three animals and confirmed the results. For physiological and behavioral experiments, replicates are the n. Replications were successful.
Randomization	Randomization was not relevant to this study. Mice of different genotypes were compared to each other.
Blinding	Immunohistological experiments, behavior and analysis were blinded. For physiological experiments and analyses was not possible to be blind because of the characteristics of the neurons.

Reporting for specific materials, systems and methods

We require information from authors about some types of materials, experimental systems and methods used in many studies. Here, indicate whether each material, system or method listed is relevant to your study. If you are not sure if a list item applies to your research, read the appropriate section before selecting a response.

Materials & experimental systems

n/a	Involved in the study	
[ ]	[x]	Antibodies
[ ]	[x]	Eukaryotic cell lines
[x]	[ ]	Palaeontology and archaeology
[ ]	[x]	Animals and other organisms
[x]	[ ]	Clinical data
[x]	[ ]	Dual use research of concern
[x]	[ ]	Plants

Methods

n/a	Involved in the study	
[x]	[ ]	ChIP-seq
[x]	[ ]	Flow cytometry
[x]	[ ]	MRI-based neuroimaging

Antibodies

Antibodies used	The following primary antibodies were used: rabbit anti-RFP (1:1000, Rockland, #600-401-379), chicken anti-RFP (1:1000, Rockland, #600-901-379), goat anti-GFP (1:1000, Rockland, #600-101-215), mouse anti-calbindin D-28K (1:10000, Swant, #CB300), guinea pig-VGAT (1:500, Synaptic Systems, #131004), guinea pig-VGLUT1 (1:2000, Merck, AB5905), guinea pig-VGLUT2 (1:2000, Merck,

	#AB2251-I), mouse-parvalbumin (1:5000, ThermoFisher Scientific, MA5-47410), and rabbit-NeuN (1:1000, Millipore, #ABN78). Secondary antibodies used in this study were: Cy3-AffiniPure Donkey anti-Rabbit (1:1000, #711-165-152), Cy3-AffiniPure Donkey anti-Chicken (1:1000, #703-165-155), Alexa Fluor 488-AffiniPure Donkey anti-Goat (1:1000, #705-545-147), Alexa Fluor 488-AffiniPure Donkey anti-Mouse (1:1000, #715-545-150), Cy3-AffiniPure Donkey anti-Mouse (1:1000, #715-165-150), Alexa Fluor 488-AffiniPure Donkey anti-Guinea Pig (1:1000, #706-545-148), Alexa Fluor 647-AffiniPure Donkey Anti-Mouse (1:1000, #715-605-151), and Cy5-AffiniPure Donkey anti-Rabbit (1:1000, #711-175-152) all from Jackson ImmunoResearch.
Validation	rabbit anti-RFP (https://www.rockland.com/datasheet/?code=600-401-379), chicken anti-RFP (https://www.rockland.com/datasheet/?code=600-901-379), goat anti-GFP (https://www.rockland.com/datasheet/?code=600-101-215), mouse anti-calbindin D-28K (https://pubmed.ncbi.nlm.nih.gov/36352508/), guinea pig-VGAT (https://sysy.com/product-factsheet/SySy_131004), guinea pig-VGluT1 (https://pubmed.ncbi.nlm.nih.gov/34038402/), guinea pig-VGluT2 (https://pubmed.ncbi.nlm.nih.gov/36352508/), mouse-parvalbumin (https://www.thermofisher.com/order/genome-database/dataSheetPdf?producttype=antibody&productsubtype=antibody_primary&productId=MA5-47410&version=Local), and rabbit-NeuN (https://www.merckmillipore.com/INTERSHOP/web/WFS/Merck-CL-Site/es_ES/-/CLP/ShowDocument-File?ProductSKU=MM_NF-ABN78&DocumentId=null&DocumentType=COA&Language=EN&Country=US&ProductBatchNo=1979271&Origin=PDP). Secondary antibodies used in this study were: Cy3-AffiniPure Donkey anti-Rabbit https://www.jacksonimmuno.com/lots/000000175018), Cy3-AffiniPure Donkey anti-Chicken (https://www.jacksonimmuno.com/lots/000000172798), Alexa Fluor 488-AffiniPure Donkey anti-Goat https://www.jacksonimmuno.com/lots/000000175407), Alexa Fluor 488-AffiniPure Donkey anti-Mouse (https://www.jacksonimmuno.com/lots/000000175021), Cy3-AffiniPure Donkey anti-Mouse (https://www.jacksonimmuno.com/lots/000000174125), Alexa Fluor 488-AffiniPure Donkey anti-Guinea Pig (https://www.jacksonimmuno.com/lots/000000173195), Alexa Fluor 647-AffiniPure Donkey Anti-Mouse (https://www.jacksonimmuno.com/lots/000000174742), and Cy5-AffiniPure Donkey anti-Rabbit (https://www.jacksonimmuno.com/lots/000000174616) from Jackson ImmunoResearch.

Eukaryotic cell lines

Policy information about cell lines and Sex and Gender in Research

Cell line source(s)	HEK293T (ATCC CRL-3216)
Authentication	The cell line was not authenticated specifically, other than confirmation that they appeared to, and grew in accordance with, published data and supplier-provided datasheets.
Mycoplasma contamination	Cells tested negative for mycoplasma contamination by PCR.
Commonly misidentified lines (See ICLAC register)	This line was commercially purchased from the indicated supplier who performed validation of cell line prior to purchase.

Animals and other research organisms

Policy information about studies involving animals; ARRIVE guidelines recommended for reporting animal research, and Sex and Gender in Research

Laboratory animals	All animals were maintained under standard temperature-controlled laboratory conditions on a 12 h:12 h light/dark cycle, with water and food available ad libitum. The following transgenic mouse lines were used in this study and maintained in a C57BL/6 background (Charles River Laboratories): Pcp2creER (Tg(Pcp2-creERT2)17.8.ICS), Ai14 (B6;129S6-Gt(ROSA)26Sortm14(CAG-tdTomato)Hze/J) (Jackson Laboratories #007908), Kir2.1 (Gt(ROSA)26Sortm2(CAG-KCNJ2/mCherry)Fmr) (kindly provided by Guillermina López-Bendito) and Pcp2cre/+ (B6.Cg-Tg(Pcp2-cre)3555Jdhu/J). For in utero electroporation surgeries, FVB/NHsd (Envigo) time-mated female mice were used and housed individually. Ages used were E12.5, P7, P14, P21-P25 and adult (P60-P90).
Wild animals	n/a
Reporting on sex	Both male and female mice were used in all experiments.
Field-collected samples	n/a
Ethics oversight	All procedures were approved by the Dutch Ethical Committee for Animal Experiments and conducted in accordance with the Institutional Animal Care and Use Committee of Erasmus Medical Center (IACUC Erasmus MC), the European and the Dutch National Legislation.

Note that full information on the approval of the study protocol must also be provided in the manuscript.

Plants

Seed stocks	n/a
Novel plant genotypes	n/a
Authentication	n/a
